# Facemask acne attenuation through modulation of indirect microbiome interactions

**DOI:** 10.1038/s41522-024-00512-w

**Published:** 2024-06-20

**Authors:** Han-Hee Na, Seil Kim, Jun‐Seob Kim, Soohyun Lee, Yeseul Kim, Su-Hyun Kim, Choong-Hwan Lee, Dohyeon Kim, Sung Ho Yoon, Haeyoung Jeong, Daehyuk Kweon, Hwi Won Seo, Choong-Min Ryu

**Affiliations:** 1 Infectious Disease Research Center, KRIBB, Daejeon, 34141 South Korea; 2 Department of Interdisciplinary Program in Biocosmetics, College of Biotechnology and Bioengineering, Sungkyunkwan University, Suwon, 16419 South Korea; 3 Division of Metrology for Quality of Life, Center for Bioanalysis, Korea Research Institute of Standards and Science, 267 Gajeong-Ro, Yuseong-Gu, Daejeon, 34113 Republic of Korea; 4 Department of Nano-Bioengineering, Incheon National University, Incheon, 22012 South Korea; 5 Functional Genomics Program, University of Science and Technology (UST), Daejeon, 34113 South Korea; 6 Department of Bioscience and Biotechnology, Konkuk University, 05029 Seoul, Republic of Korea; 7 Research Institute for Bioactive-Metabolome Network, Konkuk University, Seoul, Republic of Korea; 8 Department of Integrative Biotechnology, College of Biotechnology and Bioengineering, Sungkyunkwan University, Suwon, 16419 South Korea

**Keywords:** Microbiome, Clinical microbiology

## Abstract

During the COVID-19 pandemic, facemasks played a pivotal role in preventing person-person droplet transmission of viral particles. However, prolonged facemask wearing causes skin irritations colloquially referred to as ‘maskne’ (mask + acne), which manifests as acne and contact dermatitis and is mostly caused by pathogenic skin microbes. Previous studies revealed that the putative causal microbes were anaerobic bacteria, but the pathogenesis of facemask-associated skin conditions remains poorly defined. We therefore characterized the role of the facemask-associated skin microbiota in the development of maskne using culture-dependent and -independent methodologies. Metagenomic analysis revealed that the majority of the facemask microbiota were anaerobic bacteria that originated from the skin rather than saliva. Previous work demonstrated direct interaction between pathogenic bacteria and antagonistic strains in the microbiome. We expanded this analysis to include indirect interaction between pathogenic bacteria and other indigenous bacteria classified as either ‘pathogen helper (PH)’ or ‘pathogen inhibitor (PIn)’ strains. In vitro screening of bacteria isolated from facemasks identified both strains that antagonized and promoted pathogen growth. These data were validated using a mouse skin infection model, where we observed attenuation of symptoms following pathogen infection. Moreover, the inhibitor of pathogen helper (IPH) strain, which did not directly attenuate pathogen growth in vitro and in vivo, functioned to suppress symptom development and pathogen growth indirectly through PH inhibitory antibacterial products such as phenyl lactic acid. Taken together, our study is the first to define a mechanism by which indirect microbiota interactions under facemasks can control symptoms of maskne by suppressing a skin pathogen.

## Introduction

Since the start of the COVID-19 pandemic, wearing of facemasks has become commonplace^1^. Recent studies have demonstrated that long-term facemask wearing promoted the skin disease colloquially referred to as ‘maskne’ (mask + acne)^2–4^. Such conditions have been attributed to microbiological contamination and alteration of the skin environment such as increasing moisture and temperature caused by facemask wearing^2–4^. Microbial contamination of facemasks is a candidate risk factor for development of maskne^5–8^. For instance, following use of surgical masks, primarily by health care workers, contamination by bacteria was observed after just 10 min of use, and *S. aureus* was isolated from a mask that had been used for 2 h^9^. Bacteria and fungi have also been isolated from regular nonsurgical facemasks in use by the general population^5^. However, the role of changes to the skin microbiota in the virulence of maskne-associated pathogens remains poorly defined.

The capacity of bacteria to thrive in anaerobic conditions is indicative of their ability to cause skin infections as colonization of the anaerobic basal epithelial layer and hair follicles can promote pathogenesis and virulence^10–12^. The common skin pathogens *Cutibacterium acnes* and *Staphylococcus aureus* have the capacity to penetrate the basal epithelial layer and replicate under relatively anaerobic conditions to promote disease^13,14^. In addition, the capacity to survive in anaerobic conditions promotes evasion of the superficial epithelial defense barriers, which include dry-dead keratinized cells and tight junctions between cells^15^. These data place contamination of facemasks with anaerobic bacteria as a key risk factor for development of skin diseases including acne. In addition, previous studies demonstrated that most skin opportunistic pathogens such as *S. aureus* and *C. acnes* can grow under anaerobic conditions. Such bacteria can survive within follicles and the basal layer of epithelial cells where oxygen levels are relatively low. Thus, we hypothesized that anaerobic facemask/skin-associated bacteria generate skin-related symptoms, such as ‘maskne’^4,16^.

The microbiome plays a key role in modulating pathogen colonization, can inhibit or promote pathogen growth and associated virulence, and can also modulate host immune responses^17–19^. Mechanisms of competition between bacterial species that shape skin microbiota composition have been previously described. For instance, some strains of coagulase-negative staphylococci (CoNS) in the natural skin microbiota selectively killed *S. aureus* or *C. acnes* by secreting antimicrobial peptides such as lantibiotics, lugdunin, or phenol-soluble modulins^20–22^. Emerging evidence also indicates that CoNS can directly compete with invading skin pathogens using nonribosomal peptides. Moreover, some strains of *C. acnes* that produce thiopeptide antibiotics have been shown to reduce colonization of *S. aureus* and *S. epidermidis* in hair follicles^23^. Considering these competition mechanisms, previous studies focused on direct pathogen inhibition and development of bactericidal probiotics and prebiotics to alleviate disease symptoms^24–26^. However, such strategies based on direct pathogen inhibition have had limited success, partly due to an inadequate consideration of their effects on microbiome ecology^27,28^. The skin harbors a diverse microbiome consisting of multiple bacterial strains with the potential to interact with one another^29,30^. Moreover, recent studies highlighted complex interactions (termed higher-order interactions) involving more than single pairs of bacteria^31,32^.

Considering these complex microbial interactions which may be frequent underneath facemasks, we focused on the identification and control of pathogen helper bacterial strains that promote pathogen growth and virulence. Recent studies demonstrated that the skin pathogen *S. aureus* was frequently associated with *Pseudomonas aeruginosa* in chronic wound infections^33,34^. Co-infections resulted in worse patient outcomes than infections caused by a single pathogen^35^. These communities can display synergistic interactions that enhance virulence, persistence, or antimicrobial tolerance^36^. Taken together, it is therefore important to study not only the virulence potential of bacteria isolated from facemasks but also the interaction between pathogens and other resident bacteria.

In this study, we evaluated whether anaerobic bacteria isolated from human facemasks and skin could induce skin disease symptoms in a mouse model. Anaerobic skin bacteria were screened to identify pathogen helper and pathogen inhibitor strains which promoted and attenuated pathogen growth, respectively. Additional screening facilitated isolation of bacterial strains that inhibited growth of the pathogen helper strain and promoted growth of the pathogen inhibitor strain. Our results indicate that modulation of the pathogen helper strain could indirectly attenuate pathogen fitness resulting in reduced skin disease symptoms. This indirect pathogen inhibition strategy represents a novel therapeutic avenue which overcomes the limitations of direct pathogen inhibition.

## Results

### Microbiota analysis of facemasks, skin, and saliva

**Fig. 1:**
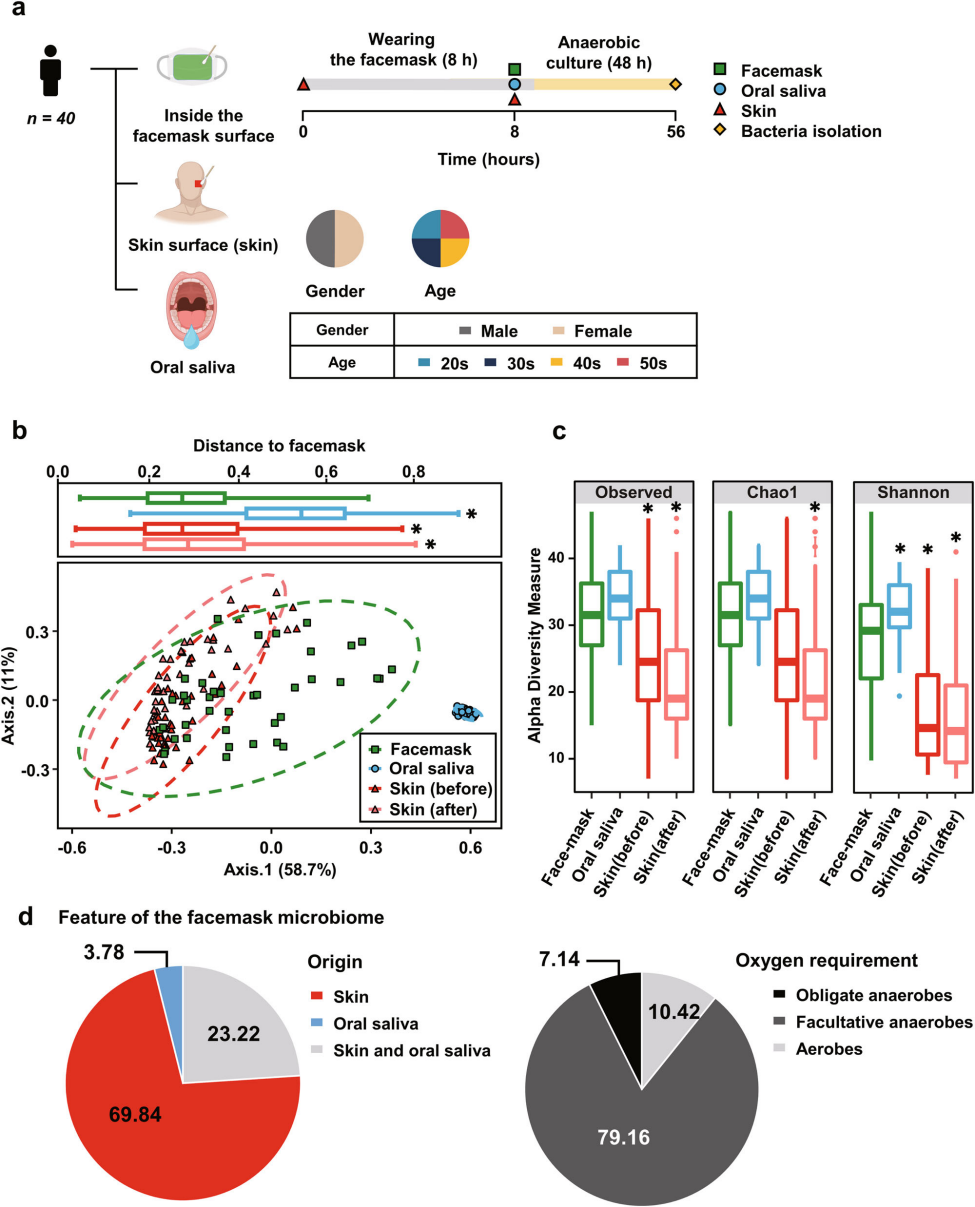
Composition of the facemask microbiome. **a** A schematic depicting experimental workflow for analysis of the facemask, skin, and saliva microbiomes. 16S rRNA amplicon sequencing. The composition of the microbiome for each sample was analyzed through 16S rRNA sequencing. This figure was generated using Biorender (https://biorender.com/). **b** Principal component analysis based on microbiome β-diversity showing separate clustering of the facemask, saliva, and skin (before and after mask wearing) microbiomes (*n* = 40). The box plots illustrate the pairwise distances of the facemask microbiome from the saliva and skin microbiomes, as calculated by the weighted UniFrac distance analysis. The median, interquartile range, minimum, and maximum are shown. The asterisks denote significant dissimilarity between the facemask microbiome and the saliva or skin microbiomes. Student’s t-test: *p < 0.05 compared with facemask microbiome. **c** Alpha diversity value distribution for indices (Observed, Chao1, and Shannon index) of the facemask, oral saliva, skin before wearing, and skin after wearing samples are shown. The line inside each box represents the median value. Outliers are shown as dots. *p < 0.05. **d** Classification of facemask microbiome origin (left). The origin of the facemask microbiome was categorized into three source patterns: skin (red), representing the proportion of facemask microbial OTUs exclusively identified in the skin microbiome; oral saliva (blue), representing the proportion of facemask microbial OTUs exclusively identified in the oral saliva microbiome; and skin and oral saliva (gray), representing the proportion of facemask microbial OTUs identified in both the skin and oral saliva microbiomes. The classification of the facemask microbiome based on their oxygen requirements at the genus level (right). The microbiome was categorized as obligate anaerobes (black), facultative anaerobes (dark gray), and aerobes (pale gray).

To analyze the composition of the facemask, skin, and saliva microbiome, we sampled 40 people of different sexes and ages (Fig. 1a). The composition of the microbiota for each sample was analyzed through 16S rRNA gene amplicon sequencing. Principle component analysis revealed that microbial community structure differed significantly between facemask, skin, and saliva microbiota (*P* < 0.05; independent, two-sided *t*-test, Fig. 1b). Weighted UniFrac distance analysis revealed that facemask microbiota were more similar to the skin microbiota than to the saliva microbiota (Fig. 1b). The facemask microbiota consisted of components of both the skin and saliva microbiota, which contributed to a significant increase in bacterial Operation Taxonomic Unit (OTU) richness in the facemask microbiome compared with that of the skin (Fig. 1c). There was no significant change to the skin microbiota before and after use of the facemask (Fig. 1b and Supplementary Fig. 1a). The skin commensals *Cutibacterium* and *Staphylococcus* were represented by more than 50% of facemask and skin microbiota sequences (Supplementary Fig. 1b). Next, to determine the origin of the facemask microbiota, OTUs within the facemask microbiome were compared to those from skin and saliva samples. Approximately 70% of the facemask microbiota originated from the skin, while less than 4% originated from saliva. The remaining 23.22% was present in both the oral and skin samples (Fig. 1d and Supplementary Fig. 1c). Facemask microbiota were further classified based on inferred oxygen requirement. Aerobes accounted for approximately 10%, and facultative and obligate anaerobes accounted for approximately 80% and 7%, respectively, of the facemask microbiome sequences (Fig. 1d and Supplementary Fig. 1d).

### Isolation of virulent skin-associated anaerobic bacteria from the facemask

**Fig. 2:**
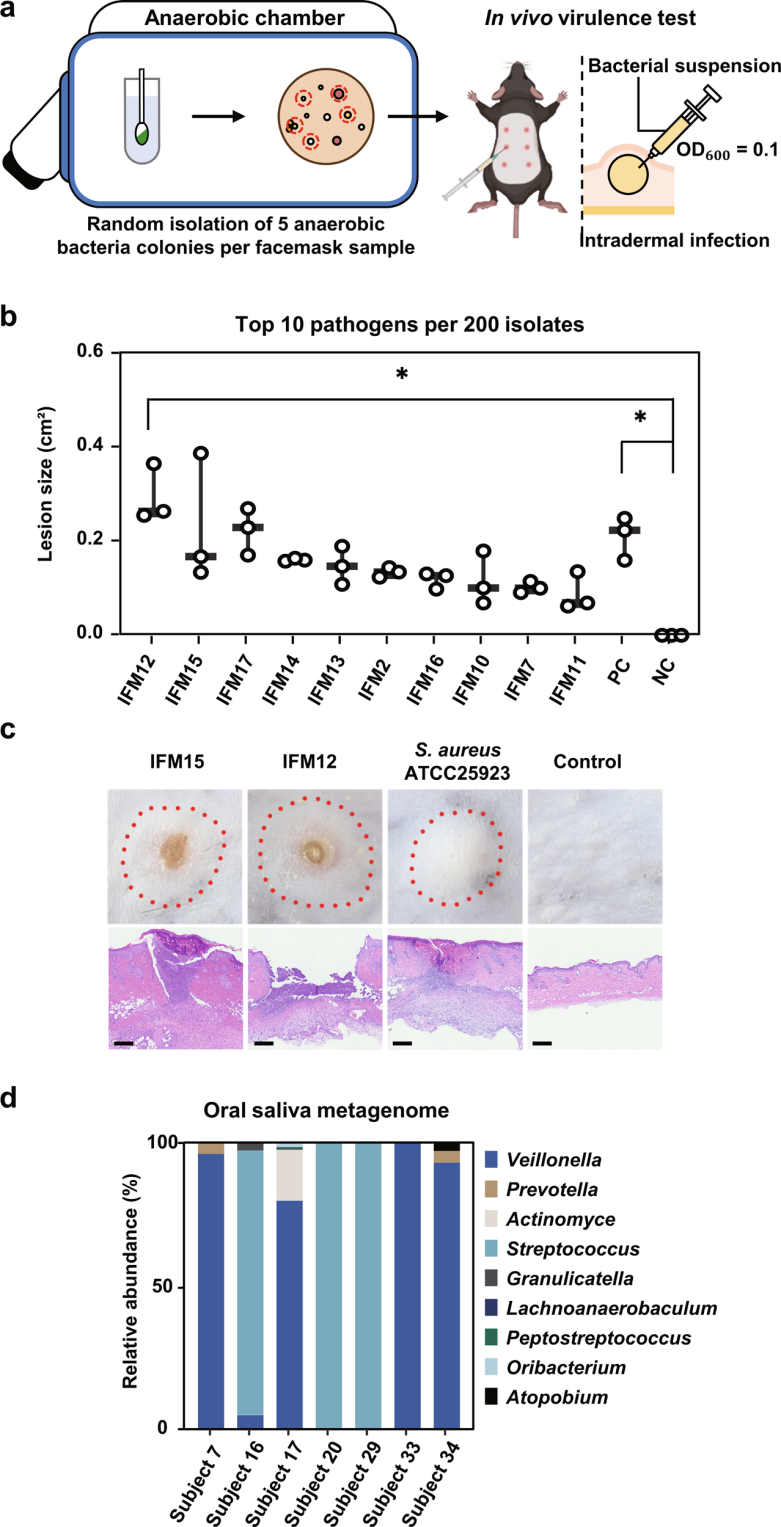
Origin of anaerobic pathogens isolated from facemasks. **a** A schematic depicting the experimental design for in vivo assessment of virulence under anaerobic conditions. Left: random isolation of five anaerobic bacteria from each facemask. Right: assessment of virulence of 200 IFM strains using an intradermal infection model. This figure was generated using Biorender (https://biorender.com/). **b** Skin lesion size following infection with IFM strains. Skin samples (*n* = 3) were evaluated 3 days after intradermal injection of IFM strains. Student’s t-test: *p < 0.05 compared with normal control (NC). The median, interquartile range, and all individual data points are shown. **c** Macroscopic (top) and microscopic (bottom) assessment of skin pathology following H&E staining. The red dotted line represents lesion area. Scale bar = 500 μm. **d** Bacterial community composition in saliva samples determined by shotgun metagenomic sequencing. The relative abundance of genus-level taxonomies is shown. The subjects were selected as seven subjects in which the top ten skin virulent IFM variants were detected.

Subsequent to the molecular analysis of the microbiome structure, we conducted a culture-dependent characterization of anaerobic bacteria present on the facemasks. We found that an average of 1.5 × 10^4^ colony forming units (CFUs) of anaerobic bacteria growing on RCM agar were present inside facemasks (Supplementary Fig. 2a). The number of anaerobic bacteria isolated from the facemasks worn by men was significantly (*p* = 0.05) higher than that isolated from the facemasks worn by women, while no significant difference was observed between age groups (Supplementary Fig. 2b). Next, to determine whether the identified anaerobic bacteria could cause inflammatory skin disease, we randomly isolated 200 bacterial strains consisting of five anaerobes per facemask sample based on colony morphology and assessed their virulence using a murine intradermal infection model (Fig. 2a and Supplementary Fig. 2c). Prior to studying the skin virulence of inside facemask (IFM) strains, we infected mice with pathogenic *S. aureus* and non-pathogenic *S. hominis* to validate that the model recapitulated human infections. Three days post-inoculation, *S. aureus* infected mice showed the widest range of skin lesions including pustules, nodules, and ulcers, while mice infected with the non-pathogenic *S. hominis* did not show any lesions throughout the duration of the experiment (Supplementary Fig. 2d). Of the 200 isolates tested, 33.5% induced skin inflammation, including pustules, nodules, and ulcers (Fig. 2b and Supplementary Fig. 2e). Among the skin virulent isolates, the lesion size induced by ten IFM strains showed no significant differences compared to that of the reference pathogen *S. aureus* ATCC 25923 (Fig. 2b, c). The top 10 skin virulent IFM strains included 5 *Staphylococcus aureus*, 3 *Staphylococcus epidermidis*, 1 *Staphylococcus capitis*, and 1 *Cutibacterium acnes* (Supplementary Table 1). Furthermore, histopathological examination showed the formation of abscesses or diffuse inflammatory cell infiltration from the epithelial to the subcutis layer following infection with virulent IFM strains (Supplementary Fig. 2f).

Next, we questioned the origin of virulent strains from IFM. We hypothesized that these strains originated from saliva and were transmitted to the facemask when speaking. To identify if the top ten skin virulent bacteria originated from saliva, we performed shotgun whole metagenomic sequencing of saliva from subjects from whom the top ten skin virulent bacteria were isolated and queried these data with the whole genome sequences of the isolated skin virulent IFM strains. The top ten skin virulent IFM strains did not match any anaerobic bacteria present in the saliva metagenome. Furthermore, no bacteria from the genera *Staphylococcus* or *Cutibacterium* were identified in the saliva metagenome (Fig. 2d). Analysis of bacterial 16S rRNA genes revealed that the genera *Staphylococcus* and *Cutibacterium* accounted for only 0.021% and 0.024%, respectively, of the saliva (oral) microbiome (Supplementary Fig. 2g). Conversely, the relative abundances of *Staphylococcus* and *Cutibacterium* were 14% and 61%, respectively, in the skin microbiome, indicating that skin virulent IFM strains were likely transferred from the skin to the facemask, rather than from the saliva (Supplementary Fig. 2g). In addition, the relative abundance of OTUs annotated as *S. aureus* in skin swabs ranging from 0.225% to 59.2% among all subjects (Supplementary Fig. 2h).

### Direct interactions between a skin virulent strain and other IFM isolates

**Fig. 3:**
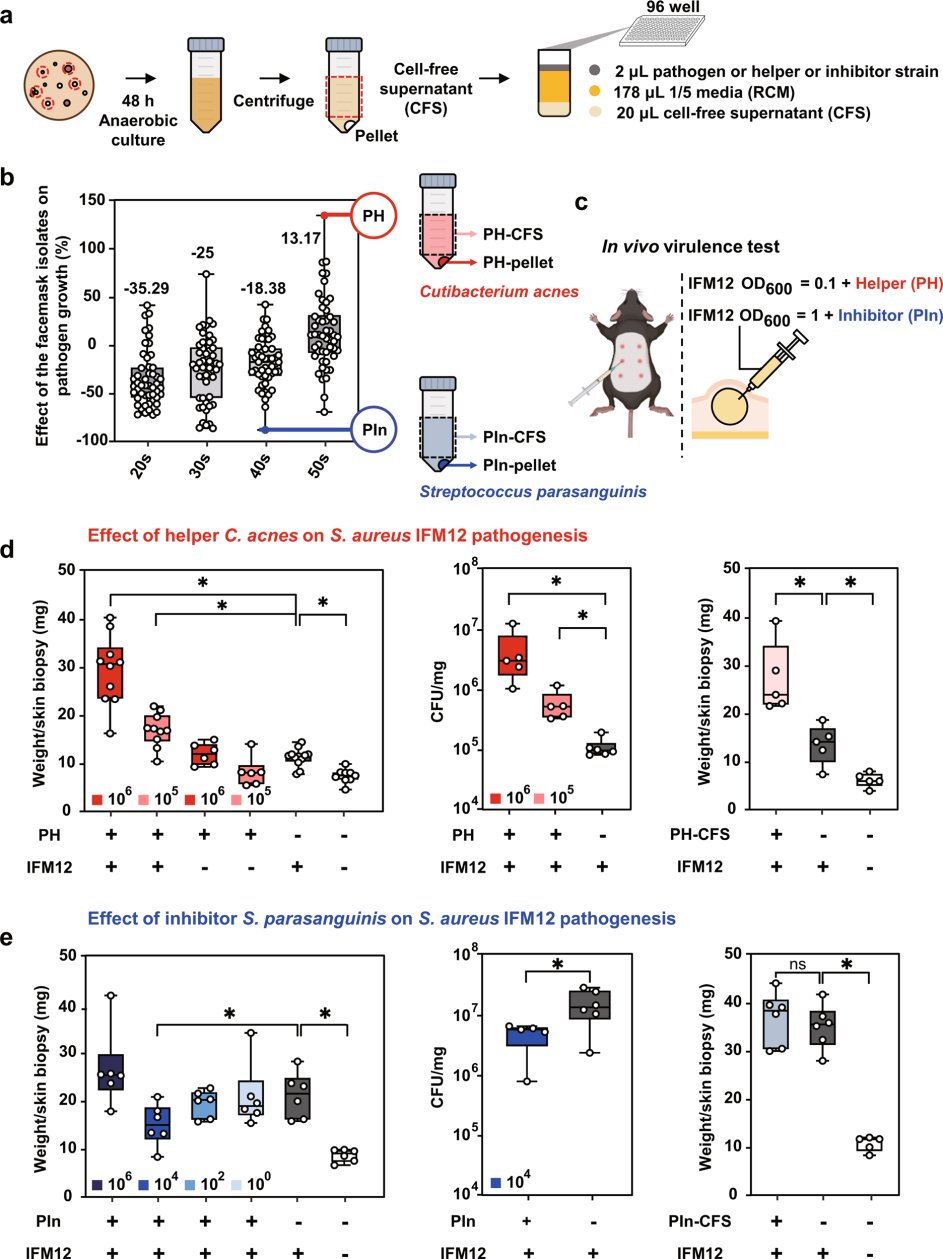
Direct interactions between skin virulent IFM and other IFM strains. **a** A schematic depicting the experimental design for screening of pathogen helper and pathogen inhibitor strains. **b** In vitro effects of CFS derived from 200 IFM isolates on IFM12 growth. The red symbol indicates the pathogen helper strain that most efficiently increased IFM12 growth, while the blue symbol represents the strains that best suppressed IFM12 growth. Box plots depicting the effects of each CFS on IFM12 growth. The number above each plot indicates the mean value. The median, interquartile range, and all individual data points are shown. **c** A schematic depicting the experimental design for assessment of pathogen virulence in vivo after co-inoculation with pathogen helper and pathogen inhibitor strains. **d** Validation of pathogen helper strains in vivo. The effect of coadministration of pathogen and either live pathogen helper strain (left panel) or cell free supernatants (right panel) on skin disease severity. Effect of live pathogen helper strains on recovery of IFM12 CFU from the skin (center panel). 10^5^ or 10^6^ indicates the CFU number of IFM12 infectious dose. The median, interquartile range, and all individual data points are shown. Student’s t-test: *p < 0.05 compared with control group infected with IFM12 alone. **e** Validation of pathogen inhibitor strains in vivo. The effect of coadministration of pathogen and either live pathogen inhibitor strain (left panel) or cell free supernatants (right panel) on skin disease severity. Effect of live pathogen inhibitor strains on recovery of IFM12 CFU from the skin (center panel). The median, interquartile range, and all individual data points are shown. Student’s t-test: *p < 0.05 compared with control group infected with IFM12 alone. Schematic representations were generated using Biorender (https://biorender.com/).

To explore interactions between a skin virulent IFM strain and other IFM isolates, we first selected the most virulent strain (IFM12) for further study (Fig. 2c). Strain IFM12 was identified as *S. aureus* based on whole genome sequencing. We evaluated the growth kinetics of strain IFM12 in cell-free supernatants (CFS) of the 200 IFM isolates to identify pathogen helper and pathogen inhibitor strains (Fig. 3a). The strains were classified as ‘pathogen helper (PH)’ and ‘pathogen inhibitor (PIn)’ strains, respectively (Fig. 3b). We further characterized the effects of the most efficient pathogen helper (Fig. 3b; red point, identified as *Cutibacterium acnes*) and pathogen inhibitor strains (Fig. 3b; blue point, identified as *Streptococcus parasanguinis*). The pathogen helper strain increased IFM12 growth by 133.35%, while the pathogen inhibitor strain decreased IFM12 growth to 88.11% compared with media-treated controls.

The top five pathogen helper and pathogen inhibitor strains were identified by 16S rRNA gene amplicon sequencing (Supplementary Table 3). The pathogen helper and pathogen inhibitor strains consisted of multiple, diverse species. Strains belonging to the genus *Cutibacterium* were identified as pathogen helpers and pathogen inhibitors (Supplementary Table 3). Among the six pathogen helper strains of *C. acnes*, five were from the IB clade, with one belonging to the IA1 clade, as determined by MLST analysis (Supplementary Table 4). The mean value of relative pathogen growth after CFS treatment from bacteria isolated from the 50 s age group was different compared with those from other age groups (Fig. 3b; *p* < 0.05). A nested ANOVA showed that both age and sex were significant factors affecting the abundance of pathogen helper strains (Table 1 and Supplementary Table 2).

**Table 1:** Factors affecting abundance of skin pathogens or pathogen helper bacteria

System	Factor	d.f.	SS	F	*P*
Pathogen effect	Age	3	9366.444	21.459	**<0.001**
	Sex	1	30.506	0.070	0.792
	Age × sex	3	527.176	1.208	0.308
Helper effect	Age	3	1053.541	7.799	**<0.001**
	Sex	1	1092.409	8.087	**0.005**
	Age × sex	3	93.833	0.695	0.556

Bold values indicate *P* < 0.05. The data obtained from results of a nested ANOVA analysis.

*d.f.* degrees of freedom, *SS* sum of squares, *F* f-statistic; *P* *p*-value.

Next, we validated the effect of pathogen helper and pathogen inhibitor strains on IFM12 pathogenesis using an intradermal mouse infection model (Fig. 3c). First, we evaluated the pathogen helper *Cutibacterium acnes* on the pathogenesis of strain IFM12. In the presence of both live pathogen helper (PH-pellet) and the pathogenic IFM12, we observed a significant increase in the weight of skin biopsies (Fig. 3d left panel) after inoculation of 10^6^ and 10^5^ CFU, and increased bacterial burdens (Fig. 3d middle panel) in infected tissue compared with pathogen infection alone. Pathogen helper CFS (PH-CFS) significantly increased the weight of infected skin biopsies (Fig. 3d right panel) compared with pathogen infection alone. Further, both PH-pellet and PH-CFS significantly exacerbated histopathological lesions, characterized by severe diffuse inflammatory cell infiltrates, epithelial erosion, and epithelial/dermal abscesses (Supplementary Fig. 3). Treatment with PH-pellet and PH-CFS in the absence of the pathogen led to normal histological findings indicating that the pathogen helper *C. acnes* is a non-pathogenic strain (Supplementary Fig. 3). Secondly, addition of the pathogen inhibitor *Streptococcus parasanguinis* significantly decreased the weight of infected skin biopsies (Fig. 3e left panel) and bacterial burdens (Fig. 3e middle panel) only at a pathogen inhibitor dose of 10^4^ CFU. These data indicate that the activity of pathogen inhibitors is dose dependent against *S. aureus* strain IFM12. Pathogen inhibitor CFS (PIn-CFS) did not significantly affect pathogen virulence (Fig. 3e right panel). Finally, we measured the activity of PH-CFS and PIn-CFS on other *S. aureus* strains including two reference strains and seven facemask-contaminating isolates. PH-CFS significantly increased the growth of strains IFM12, USA300, IFM14, IFM16, and HC10 by 46.42%, 32.5%, 20.9%, 15.5%, and 48.6%, respectively (Supplementary Fig. 4a). Conversely, PIn-CFS significantly decreased the growth of all nine *S. aureus* strains, ranging from 76.6% to 97.6% (Supplementary Fig. 4b). Taken together, these results indicate that pathogen helper and pathogen inhibitor strains identified through an in vitro interaction study can directly regulate skin pathogen virulence.

### Regulation of pathogen virulence factor expression by CFS treatment

**Fig. 4:**
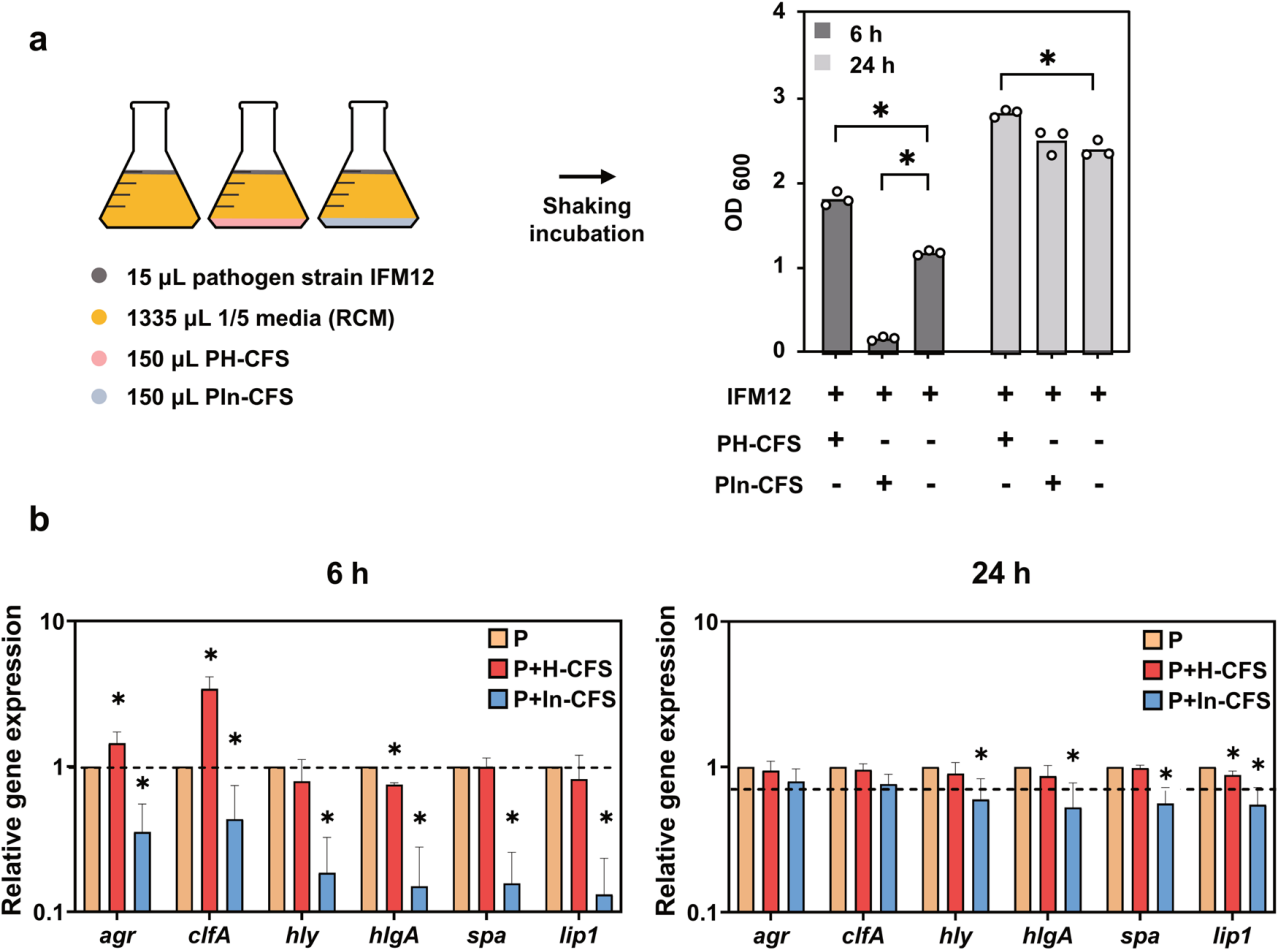
Regulation of pathogen virulence factor expression by CFS treatment. **a** Diagrammatic depiction of the workflow to study modulation of pathogen growth by pathogen helper and pathogen inhibitor strains. The bar chart shows the effects of pathogen helper strain (PH) and pathogen inhibitor strain (PIn) CFS on IFM12 growth in vitro. The mean and all individual data points are shown. Student’s t-test: *p < 0.05 compared with control group treated with IFM12 alone. **b** Changes in gene expression induced by CFS in IFM12. qRT-PCR analysis of IFM12 gene expression following treatment with PH-CFS and PIn-CFS for 6 h or 24 h. The data are expressed as the fold change in expression in treated versus nontreated IFM12. The mean and standard deviation values are shown. Student’s t-test: *p < 0.05 compared with control group treated with IFM12 alone.

To understand the mechanisms underlying attenuation of *S. aureus* IFM12 pathogenesis, we determined the effect of CFS on the expression of selected virulence and regulatory genes in *S. aureus* IFM12 by qRT-PCR (Fig. 4a). Pathogen helper strain CFS upregulated the expression of the quorum sensing regulator *agrA* and clumping factor *clfA* by 1.44-fold and 3.41-fold, respectively (Fig. 4b and Supplementary Fig. 5), after 6 h of incubation. Conversely, CFS from the pathogen inhibitor strain downregulated expression of *agrA* and *clfA* by 2.8-fold and 2.3-fold, respectively (Fig. 4b left panel). Interestingly, even after 24 h of incubation, the pathogen helper strain CFS still significantly increased the growth of *S. aureus* IFM12, though the size of effect was substantially smaller than at 6 h. Similarly, differences in expression of virulence and regulatory genes at 24 h were much smaller than at 6 h (Fig. 4b right panel).

### Screening secondary pathogen inhibitor (inhibitor of pathogen helper) strains that attenuate pathogen helper growth

**Fig. 5:**
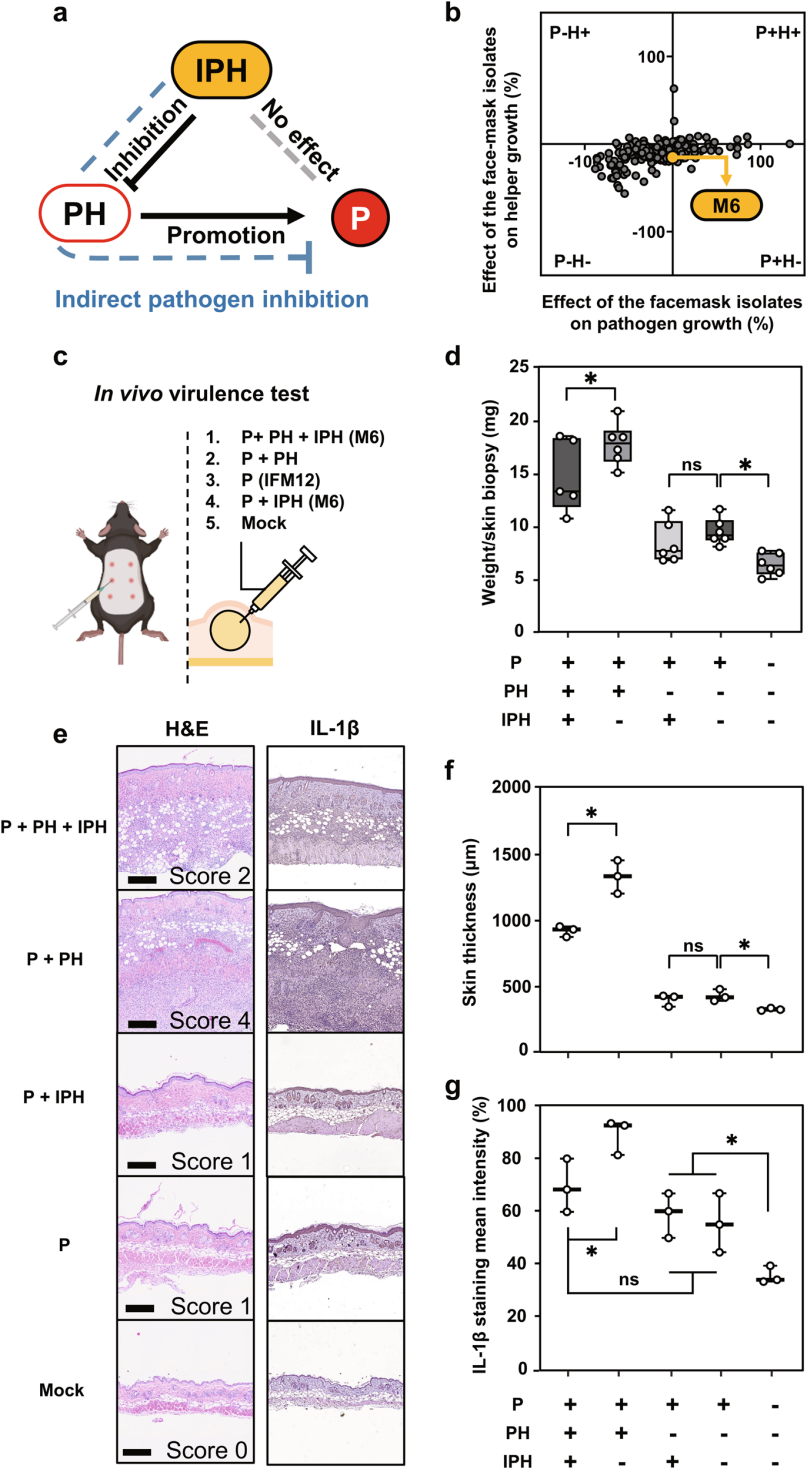
Indirect interactions between skin virulent IFM and other IFM strains. **a** A diagram depicting direct and indirect effects of pathogen helper strains on pathogen growth. Inhibitor of pathogen helper strains (IPH) refer to any IFM isolate that indirectly inhibits pathogen growth by modulating pathogen helper strains. P, pathogen; PH, pathogen helper strain; IPH, inhibitor of pathogen helper strain. **b** Effects of conditioned supernatants from 200 IFM isolates on the growth of IFM12 and the pathogen helper strain in vitro. The x-axis shows the direct effect of each IFM isolate on IFM12. The y-axis shows the effect of each isolate on the pathogen helper strain. Of the 200 IFM culture supernatants tested, M6 significantly inhibited growth of the pathogen helper strain compared with media-treated controls. **c** A schematic depicting the experimental design for assessment of pathogen virulence in vivo after co-inoculation with pathogen helper (PH) and inhibitor of pathogen helper strain (IPH). **d** Box plots depicting skin biopsy weight following pathogen infection in the presence or absence of the indirect inhibitor of pathogen helper strain M6. Skin biopsies (*n* = 3–5) with a diameter of 5 mm were taken at 6 days after intradermal injection. The median, interquartile range, and all individual data points are shown. **e** Representative skin histopathological images with H&E stains (left) and IL-1β immunohistochemistry (right). Scale bar = 500 μm. The severity of skin inflammation was determined by scoring the extent of inflammatory cell infiltration. **f** Skin thickness was measured in tissues stained with H&E. The median, and all individual data points are shown. **g** The Intensity of IL-1 β expression was quantified in IHC tissues. The median, and all individual data points are shown. One-way ANOVA with Duncan’s multiple range test: *p < 0.05. A diagram for visualization and a schematic representation were generated using Biorender (https://biorender.com/).

Following selection of pathogen helper and inhibitor strains, we investigated two means to control pathogen growth indirectly. We first attempted to re-screen and isolate a secondary helper strain acting on the pathogen inhibitor *S. parasanguinis* to indirectly attenuate *S. aureus* IFM12. Unfortunately, we failed to isolate such a strain (data not shown). Instead, we successfully identified an inhibitor of pathogen helper (IPH) strain M6 that attenuated the pathogen helper strain *C. acnes*, which subsequently indirectly inhibited *S. aureus* IFM12 (Fig. 5a). Although IPH strain M6 was identified as *S. aureus* based on 16S rRNA gene sequencing, no skin lesions developed following intradermal infection (Supplementary Fig. 6a). IPH strain M6 inhibited *C. acnes* pathogen helper growth by 13.04% without altering pathogen growth (Fig. 5b). We subsequently employed the intradermal infection model to determine whether IPH strain M6 could indirectly attenuate pathogen-induced skin lesions (Fig. 5c). When in the presence of the pathogen helper, the weight per skin biopsy increased 188.20% compared with pathogen infection alone (Fig. 5d). Treatment with CFS from IPH strain M6 decreased the weight per skin biopsy by 17.26% compared with non-CFS treatment during a pathogen and pathogen helper strain co-infection (Fig. 5d). Further, skin sample thickness significantly decreased by 30.91% in mice treated with CFS from strain M6 compared with mice infected with the pathogen and pathogen helper strain (Fig. 5e, f). IPH strain M6 CFS did not significantly alleviate symptoms when co-administered with the pathogen alone (Fig. 5f). Treatment with CFS from strain M6 resulted in a reduction of histopathological lesions and IL-1β expression characterized by inflammatory cell infiltration in the dermis and subcutaneous layer (Fig. 5e, g and Supplementary Fig. 6b). Taken together, skin symptoms were alleviated by indirectly inhibiting pathogen virulence in vivo using CFS from strain M6.

We additionally isolated strain M8 with a dual inhibitory effect on both pathogen and pathogen helper strains in vitro (Supplementary Fig. 7a, b). Similar to IPH strain M6, treatment with CFS from M8 resulted in a significant 27.93% decrease in weight per skin biopsy and a 33.39% decrease in thickness compared with non-CFS treatment in pathogen helper co-infections (Supplementary Fig. 7c–f). However, strain M8 did not decrease the weight per skin biopsy and thickness compared with single pathogen infection (Supplementary Fig. 3d–f). Moreover, we did not observe a significant difference in skin lesions between CFS treatments from strains M6 and M8. These results indicate that in vivo, IPH strains could indirectly attenuate pathogen-induced skin lesions by suppressing the pathogen helper strain.

### Identification of bacterial determinants on inhibition of pathogen helper

**Fig. 6:**
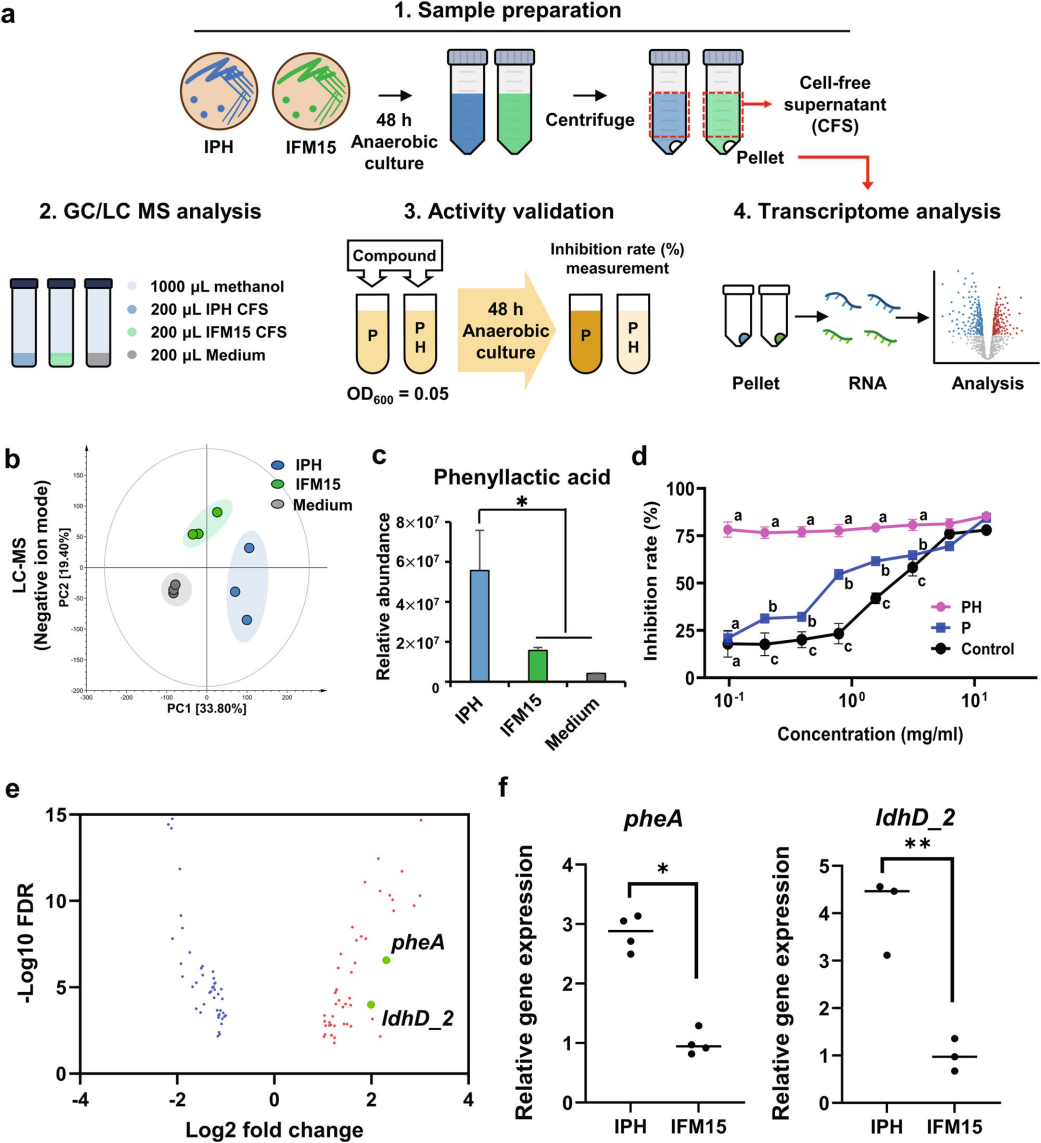
Analysis of metabolites from IPH Strain M6 with growth-inhibitory activity against pathogen helper. **a** A schematic depicting experimental workflow for analysis of the metabolite for cell-free supernatant (CFS) of IPH strain M6. IFM15, a strain with non-activity for inhibiting the pathogen helper, was used as the control. This figure was generated using Biorender (https://biorender.com/). **b** Principal component analysis based on metabolite profiles UHPLC-Orbitrap-MS datasets. **c** Bar charts show the relative abundance of phenyllactic acid derived from UHPLC-Orbitrap-MS. The IPH-specific compounds phenyllactic acid showed significantly higher concentrations in the CFS of IPH strains than in the CFS of control by fermentation. The asterisks denote significant dissimilarity between the metabolite of IPH strain and those of control. Student’s t-test: *p < 0.05. **d** Antimicrobial activity of phenyllactic acid against PH and pathogen strains was expressed as an inhibition rate (%). ANOVA followed by Tukey’s post hoc test: Different letters (a, b, and c) above the bars indicate statistically difference at *p* < 0.05. **e** Volcano plot represent the Differentially expressed genes (DEGs) between IPH and control IFM15. The genes associated phenyllactic acid biosynthesis are indicated as green circle. DEGs were selected based on |log_2_FC| > 1 and FDR < 0.05. **f** Validation for expression levels of genes associated with phenyllactic acid biosynthesis using quantitative PCR. Student’s t-test: *p < 0.05, **p < 0.01.

To identify IPH-specific compounds that inhibited the growth of the pathogen helper, we compared metabolic differences in CFS and control medium (RCM) from the IPH strain M6 and control strain IFM15 through untargeted metabolomics (Fig. 6a). The principal component analysis plot derived from GC-TOF-MS and UHPLC-Orbitrap-MS datasets showed that metabolite profiles were distinctly different between IPH and control (Fig. 6b, Supplementary Figs. 8a and 9a). The differential metabolites were selected at variable importance in the projection (VIP) > 1.0, based on partial least squares discriminant analysis (Supplementary Figs. 8a and 9a). Fifty-one metabolites were characterized using their spectrometric information (Supplementary Tables 6 and 7) and relative abundance (Supplementary Figs. 8b and 9b). Interestingly, 10 discriminant metabolites were produced by the IPH strain by fermentation at significantly higher levels relative to control. The IPH-specific compounds were 2-hydroxyisocaproic acid, 2-hydroxyisovaleric acid, phenyllactic acid, cyclo (Phe-Tyr), aureusimine A, aureusimine B, tyrosine, myo-inositol, thymine, and glycolic acid (Fig. 6c, Supplementary Figs. 8c–f and 9c–f). 2-Hydroxyisocaproic acid, 2-hydroxyisovaleric acid, and phenyllactic acid levels were 670%, 379%, and 356% higher in the IPH strain than in the control, respectively. In addition, the cyclic dipeptide cyclo Phe-Tyr was 215% higher in the IPH strain than in control. Based on these metabolomics results, we investigated the growth inhibitory effects of IPH-specific compounds against the PH strain as potential active candidates. Among the candidate metabolites, phenyllactic acid exhibited a significantly higher inhibitory rate against the PH strain (73–84%) compared to the pathogen *S. aureus* strain IMF12 (23–66%) when treated with concentrations ranging from 0.1 to 3.125 mg/ml (Fig. 6d). When treated with the same concentrations, the IPH strain used as control showed a lower inhibitory rate (10–53%) for phenyllactic acid compared to the PH strain (Fig. 6d). These results collectively support the observation that the IPH strain suppressed the growth of the PH strain without directly inhibiting the pathogen through secretion of secondary metabolites.

### Transcriptomic analysis for investigating possible metabolite synthesis pathway

To investigate possible biosynthesis pathways for metabolites that were highly produced by the IPH strain and showed antimicrobial activity against PH, we conducted a transcriptome analysis of the IPH strain M6 and control strain IFM15. Total RNAs were extracted from bacteria cultured independently for 48 h under anaerobic conditions. A total of 633 differentially expressed genes (DEGs) were identified, comprising 370 upregulated DEGs and 263 downregulated DEGs in the IPH strain. Among the 370 upregulated DEGs in the IPH strain, genes *ldhD_2* (D-Lactate dehydrogenase), *pheA* (Chorismate mutase/prephenate dehydratase), and *hisC_1* associated with the biosynthesis of phenyllactic acid, a metabolite determined by metabolomics to have inhibitory activity against the pathogen helper strain, were identified (Fig. 6e and Supplementary Fig. 10a). In a validation test using quantitative PCR (qPCR), *ldh1*, *ldh2*, *ldhD_2*, and *pheA* were found to be 13.2, 1.5, 4.0, and 2.9 times upregulated in the IPH strain, respectively, at 48 h compared to the control strain (Fig. 6f and Supplementary Fig. 10b).

## Discussion

Due to the widespread and prolonged use of facemasks, the occurrence of skin disease has increased, coining the term ‘maskne’^1,4^. Although studies have reported contamination of facemasks with bacteria^5–8^, the interactions between skin anaerobic bacteria and skin symptoms such as acne remain poorly defined. We therefore focused on bacteria cultured under anaerobic conditions to understand the interaction between maskne and the anaerobic skin microbiome, as acne can be caused by anaerobic bacteria that survive in the endodermis and skin hair follicles. We subsequently identified bacteria and their metabolites capable of suppressing opportunistic skin pathogens directly and indirectly. Our study is the first to report indirect inhibition of a target pathogen through bacterial metabolite-mediated suppression of pathogen helper growth.

We first attempted to understand the microbial dynamics of the facemask interior in the context of age, sex, and different locations including saliva, the skin surface, and the facemask surface. A prominent feature of the facemask microbiome is that the majority of contaminating bacteria originated from the skin (Fig. 2c). Around 70% of the identified OTUs in the facemask were only found in the skin, whereas only 3% were exclusively present in the oral microbiome. Bacteria from the genus *Cutibacterium*, common skin commensals, were the most abundant, comprising approximately 40% of the observed OTU. However, among previous studies investigating facemask bacterial contamination^4,6–8^, only one has reported the isolation of *C. acnes* from a facemask worn for 1 day^37^. The difference in *Cutibacterium* abundance between our study and others is likely due to differences in factors such as duration of mask wearing (4 vs. 8 h), mask type (cotton and surgical vs. quarantine), and sampling method (PBS soaking vs. swab)^7^. Furthermore, the bacterial burden contaminated in facemasks was higher in males than in females (Supplementary Fig. 2b). To the best of our knowledge, there have been no reports investigating sex differences in mask-contaminating bacteria. The difference in skin hygiene status, physiological and hormonal factors, and the use of cosmetics were considered as candidate causes of this variation. Taken together, considering the pathogenesis of facemask-induced skin disorders, alterations in the skin environment, including increased temperature and humidity due to wearing a facemask, which create optimal conditions for pathogen growth, may be major contributing factors in exacerbating skin conditions^16,38^. In addition, considering that the two genera *Staphylococcus* and *Cutibacterium* accounted for more than 50% of the bacteria present on facemasks in this study, they may also contribute to skin diseases resulting from mask contamination.

Acne and acne-like skin conditions are predominantly caused by *Cutibacterium* and *Staphylococcus*, which can colonize hair follicles and sebaceous glands under anaerobic conditions^13,39–41^. In this study, more than 30% of facemask-derived isolates caused skin inflammatory lesions of varying severity (Supplementary Fig. 2e, f). Among the anaerobically isolated bacteria, the four species *S. aureus*, *S. capitis*, *S. epidermidis*, and *C. acnes* were most common. In particular, we were able to study obligate anaerobic bacteria of the species *C. acnes*, although previous studies have excluded this species because isolation was conducted under aerobic conditions^5^. Interestingly, we found that an *S. epidermidis* strain was also virulent in the murine model (Fig. 2b and Supplementary Table 1). *S. epidermidis* is commonly considered a non-pathogenic skin commensal, but emerging studies have demonstrated that some strains are virulent^42–44^. Our data also support previous reports demonstrating a correlation between the severity of atopic dermatitis and contamination of *S. aureus* on surfaces such as beds or floors^45^. In addition, whole metagenome analysis showed that these skin virulent facemask bacteria originated from the skin rather than saliva. Considering that ~20% of healthy subjects may carry *S. aureus* in their nasal cavities^46^, we cannot exclude that the pathogenic *S. aureus* originated from nasal part. Nevertheless, additional data support that *S. aureus* is commonly isolated from the skin in healthy subjects rather than nasal (Supplementary Fig. 2h). In our experiment, including skin pathogenic carrier subjects, all subjects exhibited *S. aureus* group OTU ratios in their skin swabs ranging from 0.225% to 59.2% (Supplementary Fig. 2h). Especially, the subjects carrying the skin pathogenic *S. aureus* in facemasks showed a high relative abundance of *S. aureus* group OTU ranging from 14% to 40% in the skin (Supplementary Fig. 2h). Taken together, facemasks can act as reservoirs for skin pathogenic bacteria, and the greater the colonization of these bacteria on the facemask, the higher the likelihood of developing skin conditions.

Recent studies have demonstrated that a pathogen helper bacterium can promote target pathogen growth and virulence^47,48^. *P. aeruginosa* has been shown to enhance the virulence of co-infecting *S. aureus* in skin wounds by inducing the expression of *S. aureus* virulence factors, including Panton-Valentine leucocidin and α-hemolysin^34^. In addition, the commensal oral bacterium *Streptococcus gordonii* can enhance growth and virulence of the pathogenic co-colonizer, *Aggregatibacter actinomycetemcomitans*, by creating a high-oxygen environment, providing metabolites for pathogen growth, and establishing spatial organization in abscesses^47^. Furthermore, in cases of cystic fibrosis with polymicrobial lung infections, certain commensal *Streptococci* have been observed to stimulate the production of *P. aeruginosa* virulence factors, such as pyocyanin and elastase^48^. Such interactions play an important role in disease development under natural conditions with a diverse microbiota^24,27^. However, most studies focused on speculating the direct interaction between pathogen and counterpart and identifying individual microbes and microbial byproducts, which inhibit target pathogen(s), without considering intra- and interspecies indirect interactions in the microbiota^25,26^. In addition to direct inhibition and enhancement of pathogen growth and virulence, we speculated that an additional layer of inhibitor and helper strains could modulate pathogen growth as has been described in a previous study of the soil commensal bacteria *Ralstonia solanacearum*, which is pathogenic in *Solanaceae* family plants^27^. As we expected, our results strongly support that indirect interactions between pathogenic and commensal bacteria play an important role in the development of disease caused by skin pathogens. We therefore attempted to isolate any ‘pathogen helper’ bacteria that regulate pathogen virulence through indirect interactions and then screen bacteria that can inhibit ‘pathogen helper’ strains to indirectly inhibit pathogen growth.

First, we isolated the most effective pathogen helper strain from skin samples that modulated symptom development following *S. aureus* infection (Fig. 3b). Previous studies have reported that *C. acnes* is frequently co-isolated with *S. aureus* from infected tissue^49^. These data imply that *C. acnes* and *S. aureus* co-inhabit the skin environment, and that interaction between these two species promote disease outcome. All six pathogen helper *C. acnes* strains did not show any skin symptoms when intradermally infected alone. The lack of pathogenesis in pathogen helper *C. acnes* was also supported by MLST analysis, demonstrating that among the six *C. acnes* strains, five consisted of the IB type, which has been previously reported as the predominant commensal phylogroup^50–52^. Our data additionally revealed that *C. acnes* can not only promote *S. aureus* growth but also worsen symptoms of skin infection. Indeed, enhanced in vitro pathogen growth correlated with the outcome of an in vivo intradermal infection. We found that both CFS from pathogen helper and live pathogen helper strain exacerbated skin inflammation. This result indicates that pathogen helper strain can successfully colonize the dermis layer, leading to enhanced pathogen growth and virulence in the skin. In addition to enhanced growth, pathogen helper strain increased the virulence-related *agr* and *clfA* gene expression, which promoted pathogenesis. Furthermore, the promoting activity of the pathogen helper *C. acnes* on *S. aureus* growth was not limited to a specific IFM strain. More than 50% (4/7) of pathogenic *S. aureus* strains, including *S. aureus* USA300, which is part of the predominant highly virulent methicillin-resistant *S. aureus* clone in the United States, showed increased growth in the presence of the pathogen helper. This result suggests that pathogen helper strains could act on diverse pathogenic *S. aureus* strains and also supports the notion that pathogen helpers have the potential to exacerbate the severity of skin lesions by promoting the growth of pathogenic *S. aureus*.

Secondly, we attempted to isolate a bacterial strain and its metabolites that inhibit growth of pathogen helper strains resulting in indirect suppression of pathogen virulence. Similarly, a previous study in plants indicated that indirect inhibitory strains were effective at suppressing pathogenic *R. solanacearum* infection^27^. The mechanisms underpinning interaction between pathogen (P), pathogen helper (PH), and inhibitor of pathogen helper (IPH) strains are complex. We therefore selected IPH strain M6 that had no direct inhibitory effect on pathogen growth. Out of 200 candidate strains, the supernatant of strain M6 alleviated symptoms of intradermal infection in the mouse model (Fig. 5b–e). A *C. acnes* pathogen helper strain was not growth suppressed, compared with that of the pathogens, when incubated with CFS. This may be due to comparatively long incubation times arising from the growth rate of *C. acnes*. We found that *C. acnes*, IPH strain M6 exhibited higher production of anti-bacterial metabolites compared with the control strain. Among these metabolites, 2-hydroxyisocaproic acid demonstrated to have antimicrobial activity against obligate anaerobic bacteria, including *C. acnes* and *Porphyromonas gingivalis*, as well as multi-drug resistant *P. aeruginosa*^53,54^. In addition, phenyllactic acid (PLA) has shown broad-spectrum antimicrobial activity against *S. aureus*, *Enterococcus faecalis*, *E. coli*, *Salmonella enterica*, and *Klebsiella oxytoca* in previous studies^55,56^. Notably, phenyllactic acid (PLA, also called ‘phenyl lactate’) exhibited greater antibacterial activity against the pathogen helper, *C. acnes*, compared to the pathogenic *S. aureus* in this study (Fig. 6d). Furthermore, the upregulation of genes associated with PLA biosynthesis, including *ldh1, ldh2*, *ldhD_2*, and *pheA* (Fig. 6f and Supplementary Fig. 10b), supports the elevated production of phenyllactic acid as determined in the metabolomics analysis. Considering that PLA production has been demonstrated to be enhanced under oxygen-limiting conditions^57^, it can be inferred that interactions like those between PH and IPH strains could be effectively studied only under anaerobic conditions, as in this study. Taken together, our results support the observation that IPH indirectly alleviated pathogenic *S. aureus*-induced skin lesions through the specific inhibition of only pathogen helper *C. acnes*, without modulating the pathogen growth. In addition to the 2-hydroxy acid metabolites, cyclic dipeptides, including Aureusimines A (Tyr-Val), Aureusimines B (Phe-Val), and Cyclo Phe-Tyr, were identified as candidates for inhibiting the growth of pathogen helper. In particular, Cyclo Phe-Tyr, a diketopiperazine, was initially discovered in marine *Bacillus subtilis* and has demonstrated antibacterial activities against *S. aureus* and *S. epidermis*, with minimum inhibitory concentrations of 16 and 1 μg/mL, respectively^58,59^. Although candidate pathogen helper inhibitory metabolites produced by the IPH strain were identified, further research is required to quantify these metabolites in cell culture supernatants and skin tissue, and to evaluate their antibacterial activity. This will help clarify their biological relevance. Collectively, we consider that isolation of an IPH strain exhibiting no effects on pathogen growth provides many advantages in clinical trials. Firstly, such strains may reduce the side effects associated with direct induction of pathogen defense mechanism including development of antimicrobial resistance. Secondly, screening of further pathogen helper strains may facilitate identification of a single IPH strain capable of suppressing multiple pathogens.

One notable feature of the facemask-contaminated bacteria isolated in this study is their diverse range of activities, even among bacteria of the same species. In particular, *S. aureus* showed diverse activities depending on the bacterial strain, ranging from classification as pathogens or inhibitors of pathogen helpers. Among them, we analyzed the whole genome sequence and transcriptome of *S. aureus* pathogenic strain IFM15 and IPH strain M6. We observed that the two strains share 2361 genes, corresponding to 92–94% of the entire set of genes. Interestingly, among these 2361 shared genes, the expression levels differed significantly, resulting in 633 differentially enriched genes (DEGs). Notably, more than 30% of the DEGs were associated with the amino acid metabolic pathway. Considering that previous studies have demonstrated a link between metabolic capabilities, including amino acid biosynthesis, and pathogenicity/virulence^60^, this metabolic diversity may contribute to the strain-specific characteristics observed in facemask bacteria.

Although we provide a novel strategy to control skin disease caused by anaerobic bacterial pathogens, there are some limitations. This study did not evaluate effects on host immunity during direct and indirect attenuation of pathogen virulence. For instance, 6 days post-infection, the skin lesion induced by the pathogen alone was entirely restored by the host immune system in the skin. This restoration was impaired when the pathogen and pathogen helper were used in co-infection experiments. Extensive inflammatory cell infiltration into the dermis and hypodermis of the skin appeared to increase tissue thickness and weight^61^. These data may suggest that the host immune system becomes overwhelmed with increased pathogen growth. However, when the pathogen helper strain itself was suppressed by an inhibitor of pathogen helper (IPH) strain, disease symptoms were alleviated. These results indicate that pathogen growth can be indirectly suppressed through the action of a pathogen helper strain to improve disease outcome. To our knowledge, these data are the first demonstration of an indirect inhibition strategy for the suppression of facemask-induced skin conditions.

In conclusion, we first demonstrated presence of pathogenic anaerobic bacteria in facemasks that promote skin disease and potential control approach to reduced inflammation of the skin through indirect microbiota interaction on facial skin.

## Methods

### Ethics statement

This study was approved by the Public Institutional Review Board Designated by Ministry of Health and Welfare IRB (IRB no.: P01-202112-11-001). Each experiment was approved by the necessary Institutional Review Board, and informed consent was obtained from all participants. All methods in this study were performed in conjunction with the relevant guidelines and regulations.

### Subjects and sample collection

40 healthy volunteers of ages 20–59 years with comparable numbers of males and females were recruited. All volunteers self-reported to be in good general health with no underlying diseases. The volunteers were asked to wear a facemask for 8 h and continue their daily lives without any restriction of their behavior. Micro-organisms from the skin were sampled from the volunteers’ cheeks (4 cm^2^) with a sterile CultureSwab 220116 (BD, USA). A total of 80 skin swabs from 40 volunteers were obtained at two time points, before and after wearing a facemask. Saliva and facemask samples were also collected after wearing a facemask. The volunteers were asked to refrain from eating and drinking for 1 h prior to saliva sampling. Saliva samples (1 mL) were collected from volunteers in a 50 mL sterile Falcon tube (Becton, Dickinson and Company, New Jersey, USA). Micro-organisms from facemasks were sampled with a sterile CultureSwab 220116 (BD, USA) after removal of the facemask. All samples were stored at −80 °C before nucleic acid extraction for 16S rRNA analysis.

### DNA extraction

Genomic DNA was extracted from skin swabs, facemask swabs, and saliva samples using the DNeasy PowerSoil Kit (QIAGEN, Hilden, Germany) according to the manufacturer’s instructions. Bacterial DNA was extracted from swabs as previously described (Meisel et al., 2016). Secure PowerBead Tubes were horizontally pulsed using a Vortex Adapter for 24 (1.5–2.0 mL) tubes at maximum speed for 20 min. Tubes were placed on ice for 1 min every 10 min to prevent excess heating. Saliva samples (200 µl) were used in a single extraction, including a wash step with 1 mL TEN buffer before lysis to increase yield. Post-extraction DNA quantity and quality were measured using a Thermo NanoDrop 1000.

### PCR amplification and Illumina sequencing

PCR amplification was performed using primers targeting the V3 to V4 regions of 16S rRNA from extracted DNA. For amplification, we used primers 341F (5’-TCGTCGGCAGCGTC-AGATGTGTATAAGAGACAG-CCTACGGGNGGCWGCAG-3’; underlining sequence indicates the target region primer) and 805R (5’-GTCTCGTGGGCTCGG-AGATGTGTATAAGAGACAG-GACTACHVGGGTATCTAATCC-3’). PCR amplifications were carried out using the following conditions: initial denaturation at 95 °C for 3 min, followed by 30 cycles of denaturation at 95 °C for 30 s, primer annealing at 55 °C for 30 s, and extension at 72 °C for 30 s, with a final elongation at 72 °C for 5 min. A second amplification step for attaching the Illumina NexTera barcode was performed with a i5 forward primer (5’-AATGATACGGCGACCACCGAGATCTACAC-XXXXXXXX-TCGTCGGCAGCGTC-3’; X indicates the barcode region) and i7 reverse primer (5’-CAAGCAGAAGACGGCATACGAGAT-XXXXXXXX-GTCTCGTGGGCTCGG-3’). Conditions for secondary amplification were the same as above but with eight amplification cycles.

Successful amplification was confirmed using 1% agarose gel electrophoresis and visualization with a Gel Doc system (BioRad, Hercules, CA, USA). The amplified products were purified with the CleanPCR kit (CleanNA). Equal concentrations of purified products were pooled together, and short fragments were removed using the CleanPCR kit (CleanNA). The quality and product size were assessed using a Bioanalyzer 2100 (Agilent, Palo Alto, CA, USA) with a DNA 7500 chip. Mixed amplicons were pooled, and sequencing was carried out, at ChunLab, Inc. (Seoul, Korea) with the Illumina MiSeq Sequencing system (Illumina, USA) according to the manufacturer’s instructions.

### Processing of sequencing data

Raw sequencing data were filtering to remove low quality (<Q25) reads using Trimmomatic ver. 0.32. After QC, paired-end sequence data were merged using the fastq_mergepairs command from VSEARCH version 2.13.4 with default parameters. Primers were then trimmed with the alignment algorithm of Myers & Miller at a similarity cutoff of 0.8. Nonspecific amplicons not encoding 16S rRNA were detected using nhmmerin HMMER software package ver. 3.2.1 with hmm profiles. Unique reads were extracted, and redundant reads were clustered using the derep_fulllength command of VSEARCH. The EzBioCloud 16 S rRNA gene sequence database was used for taxonomic assignment using the usearch_global command from VSEARCH, followed by pairwise alignment. Chimeric reads were filtered for those with <97% similarity by reference based chimeric detection using the UCHIME algorithm and the nonchimeric 16S rRNA database from EzBioCloud. After chimeric filtering, reads classified to the species level (with <97% similarity) in the EzBioCloud database were compiled, and cluster_fast command was used to perform de novo clustering to generate additional OTUs. Finally, OTUs consisting of single reads were omitted from further analysis. The secondary analysis consisting of diversity calculation and biomarker discovery was conducted by in-house programs from ChunLab, Inc (Seoul, South Korea). The alpha diversity indices (ACE, Chao, Jackknife, Shannon, NPShannon, Simpson, and Phylogenetic diversity), rarefaction curves, and rank abundance curves were estimated. To visualize sample differences, beta diversity distances were calculated by several algorithms (Jensen-Shannon, Bray-Curtis, Generalized UniFrac, and Fast UniFrac). With functional profiles predicted by PICRUSt and MinPath algorithms, taxonomic and functional biomarkers were discovered by statistical comparison algorithms (LDA Effect Size - LEfSe and Kruskal-Wallis H Test). All analyses were performed in EzBioCloud 16S-based MTP, ChunLab’s bioinformatics cloud platform.

### Anaerobic culture and bacterial isolation

All culture procedures were conducted in an anaerobic chamber. The inside of the facemask swab samples were resuspended in 1 mL DPBS by vortexing. 100 µL of the facemask sample solutions was spread on an RCM agar plate and incubated for 2 days. Five anaerobic bacterial colonies were isolated per facemask sample plate. Inside facemask (IFM) strains were characterized by 16S rRNA sequencing, using the following primers:

27F 5’-AGAGTTTGATCCTGGCTCAG-3’, 1492R 5’-GGTTACCTTGTTACGACTT-3’ and 518F 5’-CCAGCAGCCGCGGTAATACG-3’ 800R 5’-TACCAGGGTATCTAATCC-3’. These two primer sets are the most basic primers used for 16S microbial identification, forming a product using the outermost primers 9F/27F or 1492R/1512R. We used these two primer sets for accurate 16S microbial identification. PCR amplifications were carried out using the following conditions: initial denaturation at 95 °C for 5 min, followed by 30 cycles of denaturation at 95 °C for 45 s, primer annealing at 55 °C for 45 s, and extension at 72 °C for 60 s, with a final elongation at 72 °C for 10 min.

### Intradermal murine infection model

Female 7–10-week-old C57BL/6J mice were purchased from Doo-Yeol Biotech and housed in randomly allocated groups of five under specific pathogen free conditions in the ABSL-2 animal facility at the Research Institute of Bioscience and Biotechnology. The experiments were conducted following the approved protocols by the Institute Animal Care and Use Committee of Korea Research Institute of Bioscience and Biotechnology (AEC-22118). All mice were provided with nesting material for enrichment. At experimental endpoints, mice were euthanized by inhalation of CO_2_ according to local guidelines. Skin infections were conducted according to previously described protocols^62,63^. Briefly, the back of each mouse was shaved with a microtome blade and wiped with alcohol pads. 30 µl of midlogarithmic bacterial suspension (OD_600nm_ of 0.1) was injected intradermally using an insulin syringe. All procedures were conducted under anesthesia via intraperitoneal injection with 200 μl of 2.5% Avertin (Sigma-Aldrich). Lesions were photographed at 3 days post-infection (DPI), and lesion areas were measured using ImageJ. At 3 DPI, mice were euthanized, and skin tissue was sampled with a 5 mm diameter biopsy and weighed. For histopathological analysis, mouse skin was flattened and fixed using 4% paraformaldehyde (PFA; Thermo Scientific, J19943-K2) for 48 h. After fixation, biopsies were embedded in paraffin and sectioned at 4 μm thickness. Biopsy sections were mounted onto slides and stained with hematoxylin and eosin (H&E) prior to analysis. Tissue slides were examined blind by a veterinary pathologist and scored from 0 to 5 as previously described. In this scale, a score of 1 indicates minimal to mild focal dermal inflammatory cell infiltrate, a score of 2 indicates mild multifocal dermal inflammatory cell infiltrate, a score of 3 indicates multifocal to diffuse moderate dermal inflammatory cell infiltrates extending to the skeletal muscle, a score of 4 indicates marked dermal inflammatory cell infiltrates extending to the skeletal muscle with intraepithelial microabscesses, and a score of 5 indicates full-thickness massive inflammation with myositis and epithelial erosion.

### Analysis of saliva anaerobic microbiota by whole-metagenome shotgun sequencing

For whole metagenome analysis, we used shotgun sequencing of saliva DNA to assess taxonomy. A total of 100 µL of saliva from the respective subject (subjects were tracked based on the presence or absence of IFM strain detection) was spread onto RCM agar plates, followed by incubation under anaerobic conditions for 48 h. Subsequently, colonies were harvested by gently spreading 100 µL of 1X PBS onto the agar surface with a spreader. Five microliters of bacterial suspension were transferred to a fresh nuclease-free tube before addition of an equal volume of 1X PBS. Ninety microliters of 40 mg/mL Lysozyme (Sigma-Aldrich) and 90 µL of 0.2 mg/mL Lysostaphin (Sigma Aldrich) were subsequently added to the tube, before incubation at 37 °C for 30 min. Subsequent steps were performed as described in the QIAamp® DNA Mini and Blood Mini Handbook under Appendix D: Protocols for Bacteria.

DNA was sequenced on an Illumina NovaSeq 6000 Sequencer by Accugene, Incheon, South Korea. Samples were processed with the Illumina NovaSeq 6000 S4 Reagent Kit v1.5 (300 cycles) according to manufacturer’s protocol.

### Analysis of IFM strains by whole genome sequencing

For whole genome analysis, we used shotgun sequencing of IFM strain DNA to assess taxonomy. DNA extraction was performed on the selected pathogenic strain, IFM, which was used for intradermal infection. Five microliters of bacterial suspension were transferred to a fresh nuclease-free tube as above before addition of an equal volume of 1X PBS. Ninety microliters of 40 mg/mL Lysozyme (Sigma Aldrich) and 90 µL of 0.2 mg/mL Lysostaphin (Sigma Aldrich) were subsequently added to the tube, before incubation at 37 °C for 30 min. Subsequent steps were performed as described in the QIAamp® DNA Mini and Blood Mini Handbook under Appendix D: Protocols for Bacteria.

DNA was sequenced on an Illumina NovaSeq 6000 Sequencer by Accugene, Incheon, South Korea. Samples were processed with the Illumina NovaSeq 6000 S4 Reagent Kit v1.5 (300 cycles) according to the manufacturer’s protocol.

### In vitro pathogen growth assays

We tested the direct effects of 200 anaerobic bacteria isolated from facemasks on the growth of IFM12 in vitro using conditioned supernatants. After 48 h of growth in RCM under anaerobic conditions at 37 °C, all bacterial cultures were centrifuged at 3000 × *g* for 5 min. Supernatants were subsequently filter sterilized with a 0.2 μm filter to remove planktonic bacteria. 20 μL of supernatant from each culture and 2 μL of OD_600_ = 0.005 pathogen culture were added to 180 μL of fresh 5x diluted RCM medium. All samples were incubated for 18 h at 37 °C with shaking before measuring the OD_600nm_ with a TECAN instrument. Statistical analysis of IFM12 growth modulation by conditioned supernatants was performed with a Bonferroni t-test comparing differences between each supernatant treatment and the control. When pathogen growth was enhanced or suppressed by a particular supernatant, the derivative strains were termed ‘pathogen helpers’ or ‘pathogen inhibitors,’ respectively. In addition, we determined whether supernatants of pathogen helper and pathogen inhibitor, obtained using the method described above, affect *S. aureus* strains other than IFM12, respectively. Two reference strains, including USA300 and ATCC25923, as well as six *S. aureus* isolates from facemasks, were incubated in RCM under aerobic conditions at 37 °C for 14 h. Subsequently, their optical density was adjusted to an OD_600nm_ of 0.05. In 96-well plates, 20 μL of supernatant from the helper and inhibitor, along with 2 μL of the pathogen culture, were added to 180 μL of freshly diluted RCM medium (5x dilution). All samples were incubated at 37 °C for 6 h with shaking, before the OD_600nm_ was measured using a TECAN instrument.

For a second round of screening, we assessed the capacity of 200 anaerobic isolates from facemasks to inhibit pathogen helper strains. We first selected strong pathogen helper strains (50M5_1), which greatly enhanced pathogen growth in supernatant assays. We then assessed whether supernatants could suppress or promote pathogen helper strain growth using the same experimental set up described above.

### Multi-locus sequence typing for *C. acnes* isolated from the facemask

Multi-locus sequence typing (MLST) analysis was conducted on pathogen helper *C. acnes* isolated from facemasks using methods outlined on the pubMLST web server (https://pubmlst.org/organisms/cutibacterium-acnes). Briefly, partial sequences of the housekeeping loci, including *aroE* (424 bp), *atpD* (453 bp), *gmk* (400 bp), *guaA* (493 bp), *lepA* (452 bp), *sodA* (450 bp), *tly* (777 bp), and *camp2* (807 bp), were PCR amplified as previously described^50,51^. PCR was carried out in a final volume of 50 μl using an AllInOneCycler™ 384-well PCR system (Bioneer, South Korea). Sequencing was performed with the BigDye Terminator v3.1 sequencing kit (Applied Biosystems, USA) on an ABI3730XL sequencer (Applied Biosystems, USA).

### In vivo assessment of pathogen virulence and skin disease development in the presence of either live pathogen helper strains or conditioned supernatants

To study the indirect effects on pathogen infection, we selected eight supernatants of isolated anaerobic bacteria from the facemask. Mouse skin was intradermally infected with the pathogenic strain in addition to the pathogen helper strain alone or together with pathogen helper supernatant. Each supernatant from the eight selected bacteria was used in combination with the strong pathogen helper strain and pathogen strain (Table S2), in addition to a PBS only control, a pathogen strain only group, and a group administered the pathogenic strain and the strong pathogen helper strain with no supernatant. The experiment was performed five times. Six days after infection, 5 mm diameter skin samples were obtained by biopsy.

### Quantification of pathogen burden

To determine pathogen infection burdens, we homogenized skin samples before serially diluting them and plating them on 1.5% agar-RCM plates containing rifampicin (50 µg/ml). Plates were subsequently incubated, and the number of colony forming units (CFU) was determined.

### Immunohistochemistry for IL-1β expression

The skin histological sections underwent a standard deparaffinization process in xylene, followed by rehydration in ethanol and blocking with 3% hydrogen peroxide. Subsequently, the sections were incubated with a polyclonal rabbit anti-IL1β antibody (NB600-633, Novus Biologicals). Following washing, the sections were subjected to a secondary antibody conjugated to horseradish peroxidase (HRP) and visualized using 3,3’-diaminobenzidine (DAB). Hematoxylin counterstaining was performed, and the signal intensity was quantified utilizing image analysis software (ImageJ 1.54e).

### Quantification of virulence gene expression of *S. aureus*

Strain IFM12 was inoculated into 15 mL of RCM broth at 37 °C in 200 mL flat bottom flasks to a starting OD_600_ of 0.05 before incubating for 6 h at 250 rpm shaking in the presence or absence of cell free supernatant (CFS). After incubation, RNase inhibitors (RNAlater, Ambion, TX, USA) were added before harvesting cells and immediately freezing on dry ice. To harvest cells, cultures were centrifuged at 15,000 × *g* for 1 min. TRIzol™ Reagent (0.75 mL) was added to bacterial pellets that had been stored at −80 °C. RNA extraction was subsequently performed according to the manufacturer’s instructions with the following modifications. To obtain RNA from *S. aureus*, we added glass beads and vortexed for 1 min, before placing the sample on ice for 1 min and repeating this process 10 times.

The RNA was dissolved in either RNase-free water for short-term usage or deionized formamide for long-term usage, and stored at −80 °C. qRT-PCR with gene-specific primers was used to determine expression of nine virulence genes (Accessory gene regulator protein A (*agrA)*, Clumping factor A *(clfA)*, Fibronectin binding protein A (*FnBPA)*, Endoproteinase Glu-C *(V8)*, Serine protease SplA*(splA)*, Zinc metalloproteinase aureolysin *(aur)*, Alpha-hemolysin *(hla)*, Transcriptional regulator (*sarA)*, and Immunoglobulin G-binding protein A *(spa*)) in IFM12 (Supplementary Table 4). qRT-PCR was performed using a SYBR Green PCR master mix (Thermo Fisher Scientific, Waltham, MA).

### Metabolite extraction

To identify IPH strain-specific compounds, we extracted metabolites from RCM medium and CFS samples from IPH and IFM15 strains. Aliquots of 200 μL were mixed with 800 μL of ice-cold methanol and vortexed for 1 min. For each sample, 800 μL was dried in a SpeedVac and was reconstituted with 1000 μL of 80% methanol. The resuspended samples were filtered through a 0.22 μm syringe filter (Hyundai Micro Co., Ltd., Seoul, Korea) for Ultrahigh performance liquid chromatography-Orbitrap-mass spectrometry (UHPLC-Orbitrap-MS) and gas chromatography-time-of-flight mass spectrometry (GC-TOF-MS) analysis.

### Mass spectrometry analysis

For GC-MS analysis, resuspended samples were dried and subjected to derivatization.

First, 50 μL of methoxamine hydrochloride in pyridine (20 mg/mL) was added to the dried samples and incubated at 30 °C for 90 min for oximation. Next, silylation was performed by adding 50 μL of MSTFA to the resulting mixture, followed by incubation at 37 °C for 30 min. The GC-TOF-MS analysis was performed using an Agilent 7890 B GC system with An Rtx-5MS capillary column (30 m × 0.25 mm × 0.25 μm particle size; Restek, Bellefonte, PA, USA) for chromatographic separation. The oven temperature was maintained at 75 °C for 2 min, before increasing to 300 °C at a rate of 15 °C/min and held for 3 min. The mass data were collected in 50–600 *m*/*z* (in positive ion mode) using Pegasus BT TOF-MS (LECO, St. Joseph, MI, USA). One microliter of the sample was injected into the GC-TOF-MS systems with a split ratio of 15:1. For LC-HRMS analysis, the UHPLC system was equipped with a Vanquish binary pump C system (Thermo Fisher Scientific) and a Waters ACQUITY UPLC HSS T_3_ column (150 mm × 2.1 mm, 1.8 μm particle size, Waters, Wexford, Ireland) for chromatographic separation. The mobile phases consisted of 0.1% formic acid in water (A) and 0.1% formic acid in acetonitrile (B). The LC eluents were run following the gradient: 5% B, 0–1 min; 5–100% B, 1–10 min; 100% B, 10–11 min; 100–5% B, 11–13 min; 5% B, 13–15 min. MS data were collected in the range of 100–1500 *m*/*z* (in negative and positive ion mode) using an Orbitrap Exploris™ 120 system combined with an ion-trap mass spectrometer (Thermo Fisher Scientific). Thirty microliters of resuspended sample was diluted with 60 μL of 80% methanol and transferred to a vial. Five microliters of the sample were injected into the LC-HRMS systems. All samples were analyzed in a random order toward non-targeted metabolite profiling.

### Data processing for metabolomics data

GC-MS raw data files were converted to NetCDF (*.cdf) using LECO Chroma TOF software (version 4.44). LC-MS raw data were converted to mzXML files using the msconvert program from ProteoWizard. The converted files of GC-MS and LC-Ms were processed for retention time correction, peak detection, and alignment using the Metalign software package (http://www.metalign.nl) and XCMS online software (https://xcmsonline.scripps.edu), respectively (HMDB; https://hmdb.ca/). Metabolic differences between IPH strains, IFM15 strains, and RCM medium were determined using SIMCA-P+ software (version 12.0, Umetrics, Umea, Sweden) based on partial least squares discriminant analysis (PLS-DA) modeling. The discriminant metabolites were selected based on variable importance in the projection (VIP) values (>1.0) and were tentatively identified by comparing in house and reference spectral libraries.

### Antimicrobial susceptibility testing

The susceptibility of pathogen helper (PH) *C. acnes* and pathogen *S. aureus* strains to phenyllactic acid was assessed using a modified version of the Clinical and Laboratory Standards Institute (CLSI) standard microdilution method. The PH and pathogen strains were cultured on agar for 2 days under anaerobic conditions. Colonies of each strain were suspended in 1 ml of RCM media with an OD of 0.3, and the suspension was further diluted at a 1:100 ratio. Subsequently, 100 µl of this diluted suspension was added to a 96-well plate. Phenyllactic acid was serially two-fold diluted in a range from 0.098 to 12.5 mg/ml and then treated and incubated with the bacterial suspension for 48–72 h under anaerobic conditions. The inhibition rate (%) was calculated by comparing the OD reduction in the treatment well to the OD of the non-treated control well.

### Determination of differentially expressed genes (DEGs) between inhibitor of helper bacteria (IPH) and non-IPH strain

The quality of RNA sequencing raw data was checked using FastQC (v0.11.9) software (2015), after the trimmed using Trimmomatic (v0.39) software. The trimmed data were mapped to the whole genome sequences of each strain using bowtie2 (v2.4.5) software. The mapping data were assigned read counts to each gene using HTSeq (v1.99.2). To compare transcription levels between strains, an orthologous search was performed using OrthoFinder (v2.5.5) software, and the control *S. aureus* IFM15 gene with the highest score was assigned to each gene of IPH strain M6^64^. Afterwards, filtering and trimmed mean of M-values (TMM) normalization were performed using the edgeR (v3.36.0) package of the R (v4.1.3) language, and Differentially expressed genes (DEGs) were selected based on |log2FC| > 1 and FDR < 0.05. Cluster of orthologous groups (COGs) were assigned to the determined DEGs using a conserved domain search service^65^. Afterwards, hypergeometric *p*-values were obtained for the designated functional pathway associated with the COG, and these *p*-values were adjusted with Bonferroni correction. Significant functional pathways were selected based on these *p*-value being less than 0.05.

### Statistical analysis

All descriptive data is presented as median with interquartile range. The skin virulence of the facemask isolates and the effect of pathogen helper and inhibitor on pathogen growth and skin lesion were analyzed by Student’s *t*-test. When comparing mean differences between groups to evaluate indirect interactions between the skin virulent inside facemask strain (IFM) and other IFM strains, we used an analysis of variance (ANOVA) and Duncan’s multiple range test. Differences were considered significant when *p*-values were less than 0.05. When comparing mean differences between groups for evaluation of indirect interactions, we used an analysis of variance (ANOVA) and Duncan’s multiple range test. Differences were considered significant when *p*-values were less than 0.05. Statistical analyses and graphs were performed using GraphPad Prism version 10. The relationship between age and direct/indirect effects on pathogen strain growth in vitro was analyzed using linear regression using R 3.6.3 (www.r-project.org) and Sigma Plot (V.12.5). Metabolic difference between IPH strains, IFM15 strains, and RCM medium were considered significant based on ANOVA and Duncan’s multiple range test, and also by Student’s t-test for IPH-specific compounds.

### Supplementary information

Supplementary Tables and Figures

Reporting summanry

## Data Availability

The raw 16S rRNA gene sequence data produced as part of the present work were deposited at the NCBI SRA database (Accession number: PRJNA1058139). In addition, RNA-seq data were deposited at the NCBI GEO database (Accession number: GSE248604).
