# Supplementary material for: Facemask acne attenuation through modulation of indirect microbiome interactions
Source: NPJ Biofilms Microbiomes. 2024 Jun 20;10:50. doi: 10.1038/s41522-024-00512-w (PMC11190265; doi:10.1038/s41522-024-00512-w)
Supplement: Supplementary file 1 — Supplementary Tables and Figures [file 41522_2024_512_MOESM1_ESM.pdf]

Supplementary Tables

Supplementary Table 1. List of the top 10 pathogens of the 200 isolates

Virulence rank	ID	Species	Origin
1	IFM12	*Staphylococcus aureus*	Subject 20
2	IFM15	*Staphylococcus aureus*	Subject 33
3	IFM17	*Staphylococcus capitis*	Subject 7
4	IFM14	*Staphylococcus aureus*	Subject 33
5	IFM13	*Staphylococcus aureus*	Subject 20
6	IFM2	*Cutibacterium acnes*	Subject 16
7	IFM16	*Staphylococcus aureus*	Subject 33
8	IFM10	*Staphylococcus epidermidis*	Subject 29
9	IFM7	*Staphylococcus epidermidis*	Subject 17
10	IFM11	*Staphylococcus epidermidis*	Subject 34

IFM: inside facemask strains.

Supplementary Table 2. Correlation analysis of age, direct, and indirect effects

**Factor**	**R**	***P*-value**
Age vs. direct effect	0.467	<0.01
Age vs. indirect effect	-0.022	0.755
Direct effect vs. indirect effect	-0.082	0.248

P, two-sided Fisher's exact or Mann–Whitney U test. Bold values indicate *P*<0.05.

Supplementary Table 3. List of the top 5 pathogen helper and pathogen inhibitor strains isolated from facemasks

No.	IFM number	Helper species	Effect of pathogen growth (%)	No.	IFM number	Inhibitor species	Effect of pathogen growth (%)
H1	50M5_1	*Cutibacterium acnes*	133.35	In1	40F1_5	*Streptococcus parasanguinis*	-88.11
H2	50F3_5	*Staphylococcus caprae*	85.66	In2	30M3_3	*Cutibacterium avidum*	-86.35
H3	50M4_3	*Cutibacterium acnes*	84.83	In3	30M3_4	*Cutibacterium avidum*	-85.82
H4	50F4_1	*Staphylococcus aureus*	73.84	In4	30M3_5	*Cutibacterium avidum*	-83.14
H5	50M4_2	*Cutibacterium acnes*	73.55	In5	30M1_5	*Cutibacterium avidum*	-82.15

List of the top 5 pathogen helper and pathogen inhibitor anaerobic bacterial strains, indicating the percentage effect on growth compared with the control. %: percentage effect on pathogen growth.

Supplementary Table 4. Phylotyping[a] of pathogen and pathogen helper *Cutibacterium acnes* strains isolated from the facemask

Strain	IFM number	Species	ST[b]	Clade	Skin pathogenicity
HC[c] 1	20F1_3	*Cutibacterium acnes*	1	IA1	None
HC2	20F1_4	*Cutibacterium acnes*	53	IB	None
HC5	50M4_1	*Cutibacterium acnes*	5	IB	None
HC6	50M4_2	*Cutibacterium acnes*	5	IB	None
HC7	50M4_3	*Cutibacterium acnes*	5	IB	None
HC9	50M5_1	*Cutibacterium acnes*	5	IB	None
IFM2[d]	30F1_1	*Cutibacterium acnes*	5	IB	Yes

[a]Phylotype was determined by MLST analysis based on sequencing of *aroE*, *atpD*, *gmk*, *guaA*, *lepA*, *soda*, *tly*, and *CAMP2* genes.
[b]Sequence type.
[c]Pathogen helper strain.
[d]Skin pathogenic strain.

Supplementary Table 5. PCR primers used in this study.

Gene	Primers	Primer sequences	Notes
gmk	gmk F	AGTTTAGATCACTTGAGAGAGCGA	*S. aureus* housekeeping gene
	gmk R	TCATTTCAACTTCTTTACGCGCTT	
gyrB	gyrB F	AATGGTCTATTGCGCGATACAAAG	housekeeping gene
	gyrB R	CGCTTCTATCGCATCTTCAAGTTT	
agrA	agrA F	CTCGCAACTGATAATCCTTATGAGG	
	agrA R	GTAACGAAAATAATGTTACCAACTGGG	
clfA	clfA F	CAACGAATCAAGCTAATACACCG	
	clfA R	GTTGTTGAAACATTTTCCGCATTTG	
hly	hly F	TATTGGTGCAAATGTTTCGATTGG	
	hly R	TCTGCTGCTTTCATAGAACCATTT	
hlgA	hlgA F	AAAGCAAATTCATTCGTTACACCG	
	hlgA R	CGTAAGCATATGTAGCATCCATGT	
spa	spa F	AACACGATGAAGCTCAACAAAATG	
	spa R	TGTTGAAGTTATTTTGTTGCGCAT	
lip1	lip1 F	TTACCAAACAAAGGGACAAAGGAA	
	lip1 R	CGAATGTTCATTTTATTACCGCCC	
sarA	sarA-F	GAGTTGTTATCAATGGTCACTTATGCTG	
	sarA-R	CTTTGTTTTCGCTGATGTATGTCAATAC	
hla	hla-F	TATAGTCAGCTCAGTAACAACAACA	
	hla-F	TGCATGCCATTTTCTTTATCATAAGTGAC	
fnbA	fnbA-F	ATTGAAACAATAGAAGAAACGGATTCATC	
	fnbA-F	CTTCAAAGTCAATTGGATTTGATTCCTC	
traP	traP_F	TGCAATCTTCATTCCATCAACAGA	
	traP_R	ATGTCGATCAGCAAATCCGAAATA	
cap8	cap8_F	AGTGGTCATAAACAAGATTCGGAA	
	cap8_R	TGCACCGATTAGATTCACTACAGT	
V8	V8_F	CAGGCGAAGGTGATTTAGCAATAG	
	V8_R	CATATTGCATAGCTTCGCCTTTGA	
FnBPB	FnBPB_F	GCATACGTGAAACCTAACAACCAA	
	FnBPB_R	CACACTTTCCGCTAAACCTTCTTT	
IsdA	IsdA_F	ACACCTACTGAGCAAACTAAACCA	
	IsdA_R	TGATTGTTGCTTTCAGATTTCGCT	
sspB	sspB_F	CAATTCGATAACTCATGGTGTGCA	
	sspB_R	GTCTTGCTCACTTACTTCAGGGTA	
scpA		AAGCTTCTGTCACTATGCCAACTA	
	scpA_F		
	scpA_R	AATCGTTGTCCTTGTAAGTTTGGG	

hysA	hysA_F	TGAAAATGCCTATGGAAAAGAGCC	
	hysA_R	TCACTATCTGGGGCGAAAGTTTTA	
GAPDH	GAPDH-F	TGATGACATCAAGAAGGTGGTGAAG	
	GAPDH-R	TCCTTGGAGGCCATGTAGGCCAT	
aroE	aroE_F	GTGATTGGCCATCCAGTG	C. acnes phylotyping primers
	aroE_R	CGCTGTGGACCTCAAAAC	
atpD	atpD_F	AATTACCCCCGAGACGAA	
	atpD_R	CGTGTTCTGGGACAGGAA	
gmk	gmk_F	TAGCCATCCGGAGATCGT	
	gmk_R	GCGCAACTGCGTGATCTA	
guaA	guaA_F	TCGCCTTCATGGAACAAC	
	guaA_R	CCATAAGTACGCCCGTCA	
lepA	lepA_F	TCGCGCCCAGTACTTAGA	
	lepA_R	CGGATTTCCACTCGATCA	
sodA	sodA_F	TGGAACTGCACCATGACA	
	sodA_R	GCTAACGACGTTCCACCA	
tly	tly_F	CAGGACGTGATGGCAATGCGA	
	tly_R	TCGTTCACAAGACCACAGTAGC	
CAMP2	CAMP2_F	GTCGTAGCCATACACCACACG	
	CAMP2_R	GCACCGAGTGTTGATGTCAATTAGC	

Supplementary Table 6. List of discriminant metabolite (VIP>1.0) and their spectral characteristics based on GC-TOF -MS.

No.	Tentative identification	VIP[a] 1	VIP 2	*p* value	RT[b] (min)	Identified ion (m/z)	Mass fragment[c]	TMS
**Amino acid and derivatives**								
1	Alanine	0.02	1.34	0.02	5.82	116	116, 73, 117, 74, 103, 118, 100	2
2	Valine	0.22	1.33	0.00	7.01	144	144, 218, 145, 100, 146, 72, 219	2
3	Leucine	0.37	1.23	0.00	7.56	158	158, 159, 102, 160, 100, 86, 232	2
4	Glycine	0.47	1.68	0.00	7.92	174	174, 86, 175, 73, 248, 100, 59	3
5	Aspartic acid	1.17	1.49	0.03	9.78	232	73, 232, 100, 218, 147, 233, 74	3
6	Phenylalanine	0.17	1.39	0.02	10.08	120	120, 146, 75, 91, 130, 121, 103	1
7	Tyrosine	0.60	1.68	0.00	12.62	179	179, 180, 308, 181, 309, 310, 281	2
**Organic acid**								
8	2-Hydroxybutyric acid	0.19	1.48	0.00	6.08	131	75, 73, 131, 147, 58, 132, 148	2
9	Oxalic acid	0.62	1.48	0.00	6.12	190	73, 75, 52, 147, 51, 148, 190	2
10	3-Hydroxybutyric acid	0.62	1.20	0.04	6.42	191	147, 117, 75, 191, 148, 233, 66	2
11	Succinic acid	1.22	1.44	0.01	7.93	247	147, 75, 148, 247, 55, 149, 56	2
12	Glyceric acid	1.27	1.55	0.00	8.13	292	73, 147, 189, 103, 133, 292, 102	3
13	Malic acid	1.20	1.57	0.00	9.51	233	73, 147, 75, 233, 55, 74, 133	3
14	2-Hydroxyglutaric acid	1.22	1.56	0.00	10.20	247	129, 75, 247, 147, 157, 85, 203	3
15	D-Gluconic acid	1.72	1.24	0.00	13.39	333	73, 147, 205, 103, 333, 292, 217	6
**Sugar and derivatives**								
16	Glycolic acid	0.79	1.29	0.00	5.51	205	73, 147, 66, 148, 74, 205, 177	2
17	D-Fructose	0.84	1.30	0.00	12.50	217	73, 103, 217, 307, 147, 74, 75	5

18	D-Glucitol	1.69	1.21	0.00	12.90	319	73, 205, 147, 319, 103, 217, 117	6
19	Myo-Inositol	1.67	1.31	0.00	13.94	305	73, 217, 147, 305, 191, 318, 204	6
20	Sucrose	1.81	1.34	0.00	17.01	362	73, 217, 362, 73, 147, 361, 103	8
21	Lactose	1.81	1.32	0.00	17.56	361	73, 361, 191, 217, 147, 362, 103	8
**Nucleotides**								
22	Uracil	1.21	1.57	0.00	8.22	241	241, 99, 73, 147, 256, 255, 113	2
23	Thymine	1.27	1.55	0.00	8.79	255	255, 73, 113, 270, 147, 256, 120	2
24	Uridine	0.88	1.20	0.00	15.98	217	73, 217, 103, 147, 75, 169, 218	3
**Alcohols**								
25	2,3-Butanediol	0.06	1.38	0.00	5.11	117	117, 73, 147, 118, 75, 74, 66	2
26	Triethylene glycol	0.13	1.72	0.06	9.61	117	73, 117, 116, 103, 101, 75, 161	2
**Inorganic compound**								
27	Hydroxylamine	1.24	1.55	0.04	5.96	249	73, 133, 146, 119, 59, 249, 86	3
28	Phosphoric acid	1.52	1.46	0.05	7.60	299	299, 300, 314, 301, 133, 193, 207	3

[a]VIP, variable in projection;
[b]RT, retention time;
[c]TMS, number of trimethylsilyl groups.

Supplementary Table 7. List of discriminant metabolite (VIP<1.0) and their spectral characteristics based on UHPLC-Orbitrap-MS.

No.	Tentative identification	VIP1	VIP2	p-value	RT (min)	$[M-H]^-$	$[M+H]^+$	M.W.	Molecular formula	Error (ppm)	MS^2 fragment pattern (*m/z*)
Amino acids and peptides											
1	5-Hydroxylysine	1.01	1.09	0.12	0.67	161.0930	163.1073	162	C6H15N2O3	-2.85	(+)163>128, 82
2	Ala-Lys	1.90	1.40	0.00	0.65	216.1348	218.1493	217	C9H20N3O3	-2.66	(+)218>147, 129, 84
3	Glu-val	1.43	1.32	0.00	1.08	245.1136	-	246	C10H18N2O5	-2.64	(-)245>227, 130
4	Leu-Val	1.77	1.44	0.00	1.16	229.1553	231.1698	230	C11H23N2O3	-2.31	(+)231>86, 72
5	Thr-Phe	1.81	1.47	0.00	1.26	265.1190	267.1333	266	C13H19N2O4	-2.37	(+)267>166, 74, 120
6	Ile-Leu	1.82	1.47	0.00	5.44	243.1709	245.1857	244	C12H25N2O3	-1.22	(+)245>144, 132, 86
7	Acetyl-phenylalanine	1.35	1.01	0.00	6.37	206.0820	208.0966	207	C11H13NO3	-1.44	(-)206>164, 147, 91
8	Ile-Ile-Pro	1.86	1.32	0.00	5.98	340.2231	342.2380	341	C17H32N3O4	-2.03	(+)342>183, 70, 211, 86
9	PyroGlu-Phe	1.14	0.94	0.21	6.02	275.1031	277.1178	276	C14H15N2O4	-1.75	(+)277>231, 166, 120, 84
10	Lactoyl-leucine	0.42	1.56	0.00	6.19	202.1081	204.1226	203	C9H17NO4	-1.69	(-)202>158, 130
11	Aureusimine A	1.04	1.33	0.00	6.22	-	245.1281	244	C14H16N2O2	-1.27	(+)217, 154, 120
12	Cyclo (Phe-Tyr)	1.57	1.49	0.00	6.44	309.1240	311.1383	310	C18H19N2O3	-2.19	(+)311>136, 120, 107
13	Lactoyl-phenylalanine	0.65	1.56	0.00	6.51	236.0924	238.1068	237	C12H15NO4	-1.80	(-)237>145, 101, 88
14	Cyclo (Trp-Pro)	0.99	1.02	0.17	6.64	-	284.1387	283	C16H18N3O2	-2.18	(+)284>130, 170, 97
15	Aureusimine B	1.69	1.30	0.03	8.49	-	229.1329	228	C14H16N2O	-2.91	(+)229>214, 159
16	Dipeptide 1	0.78	1.55	0.00	0.68	225.0988	227.1132	226	C9H14N4O3	-2.21	(-)225>154, 110, 88
17	Dipeptide 2	0.55	1.59	0.00	0.68	239.1143	241.1288	240	C10H16N4O3	-2.38	(-)239>195, 168, 87
2- Hydroxy acids											
18	Phenyllactic acid	1.53	1.12	0.00	6.33	165.0554	-	166	C9H10O3	-1.71	(-)147, 119

19	Hydroxyphenyllactic acid	1.64	1.22	0.00	1.73	181.0502	-	182	C9H10O4	4.02	(-)163, 135, 119
20	2-Hydroxy isovaleric acid	1.15	1.18	0.00	2.11	117.0557	-	118	C5H10O3	-0.46	(-)99, 71
21	2-Hydroxy isocaproic acid	1.23	1.34	0.00	5.78	131.0713	-	132	C6H12O3	-0.60	(-)85
Fatty acids and Lipids											
22	Azelaic acid	0.45	1.00	0.24	6.92	187.0972	-	188	C9H15O4	2.50	(-)187>125, 97
23	Glycochenodeoxycholic acid	1.07	1.09	0.064	9.17	448.3056	-	449	C26H42NO5	2.93	(-)448>404, 73

[a]VIP, variable in projection;
[b]RT, retention time;
[c]M.W., molecular weigh

Supplementary figures

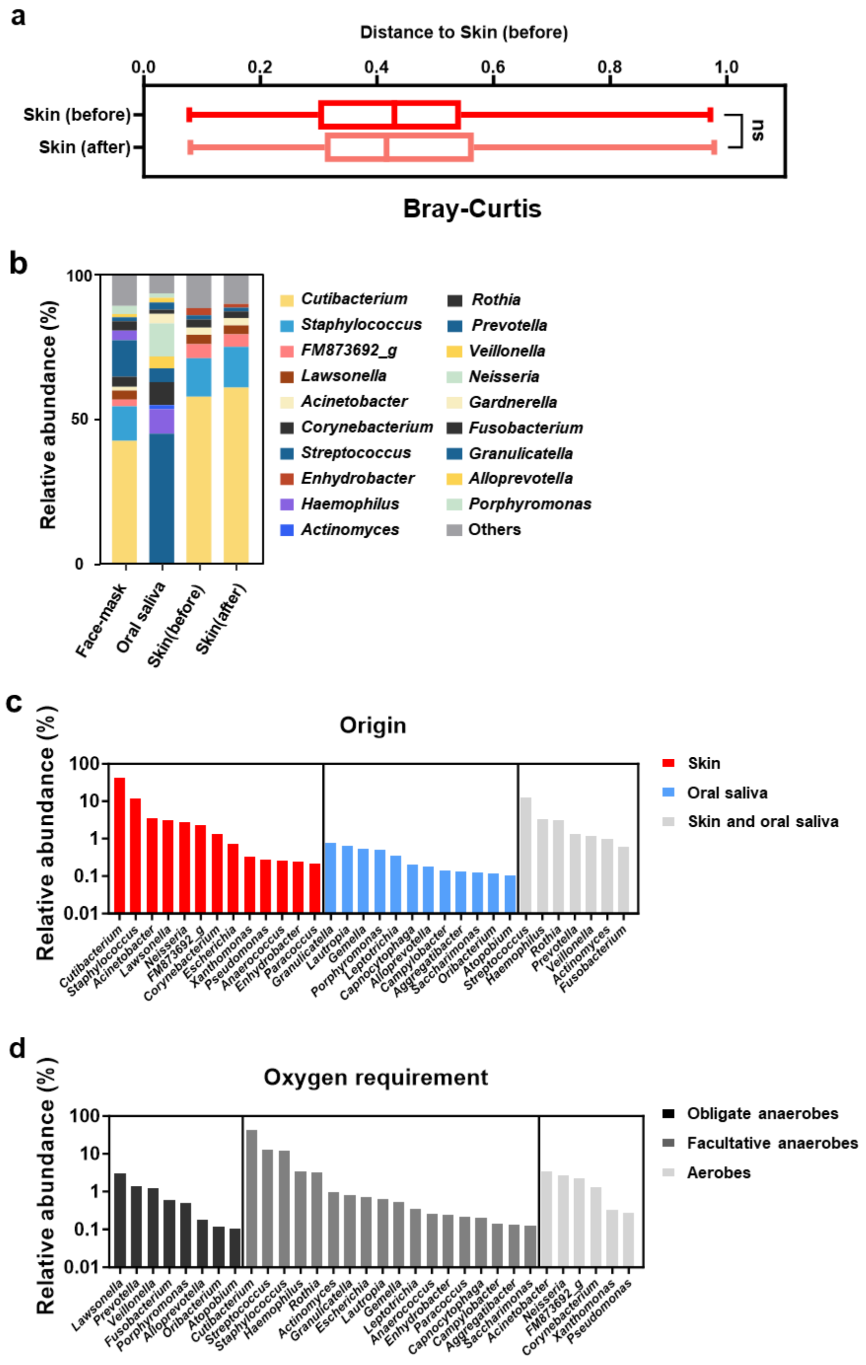

Supplementary Figure 1: Microbiome analysis of facemasks

a The box plots illustrate the Bray-Curtis distance between the skin microbiome before and after wearing a facemask. **b** The relative abundance of taxa at the genus level. Relative abundance of the dominant genus in the microbiome of all groups (the facemask, oral saliva, skin before wearing, and skin after wearing). **c** Relative abundance of bacterial genera in facemasks, grouped by origin. The red bar graph

represents microbiota derived from the skin, the blue bar graph represents microbiota derived from the oral cavity, and the gray bar graph represents microbiota originating from both the skin and oral cavity. **d** Relative abundance of bacterial genera in facemasks, grouped by oxygen requirement. The microbiome was categorized as obligate anaerobes (black), facultative anaerobes (dark gray), and aerobes (pale gray).

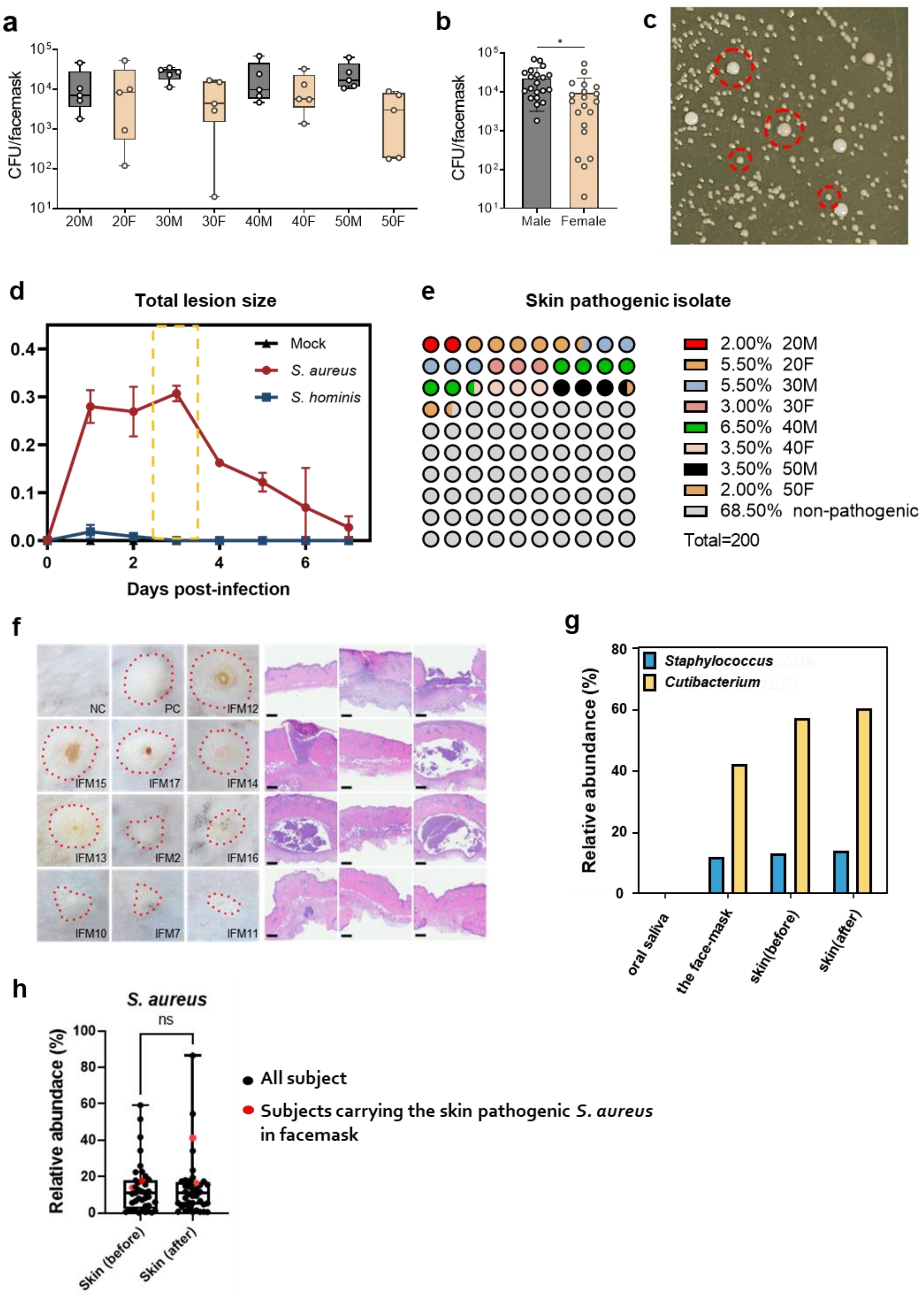

Supplementary Figure 2: Pathogenic evaluation of bacteria isolated from facemasks

a Colony forming units of anaerobic bacteria from inside the facemask. The x-axis represents the sex and age of the cohort. Each point represents an individual value of the inside facemask bacterial burden; the mean value is represented by the horizontal line. Error bars represent SEM (n=5). The data were analyzed using a one-way ANOVA comparing 3 or more groups. **b** The x-axis represents the sex of the cohort, with a statistically significant difference in the relative abundance of anaerobic bacteria in facemasks based on

sex. * $p < 0.05$**.** **c** An image displaying the appearance of anaerobic bacterial colonies isolated from facemasks. **d** Representative mean total skin inflammation lesion size (cm^2) ± SEM. Based on these results, we compared and analyzed the lesion sizes at 3 DPI in subsequent experiments. **e** The relative abundance of *Staphylococcus* and *Cutibacterium* in all groups (oral saliva, the facemask, skin before wearing, and skin after wearing). **f** Skin samples (n=3) were evaluated at 3 DPI with IFM isolates. Skin macroscopic (left panel) and microscopic (right panel) observations**. g** Ratio of skin pathogenic bacteria representing the proportion of bacteria causing skin diseases among the total 200 isolated strains. Among the 200 strains, 63 isolates (33.5%) were confirmed to be pathogenic to the skin. **h** Relative abundance of *S. aureus* group OTU in microbiome analysis for skin swab of all subject. The red circle indicates the results corresponding to subjects carrying the skin pathogenic *S. aureus* on the facemask.

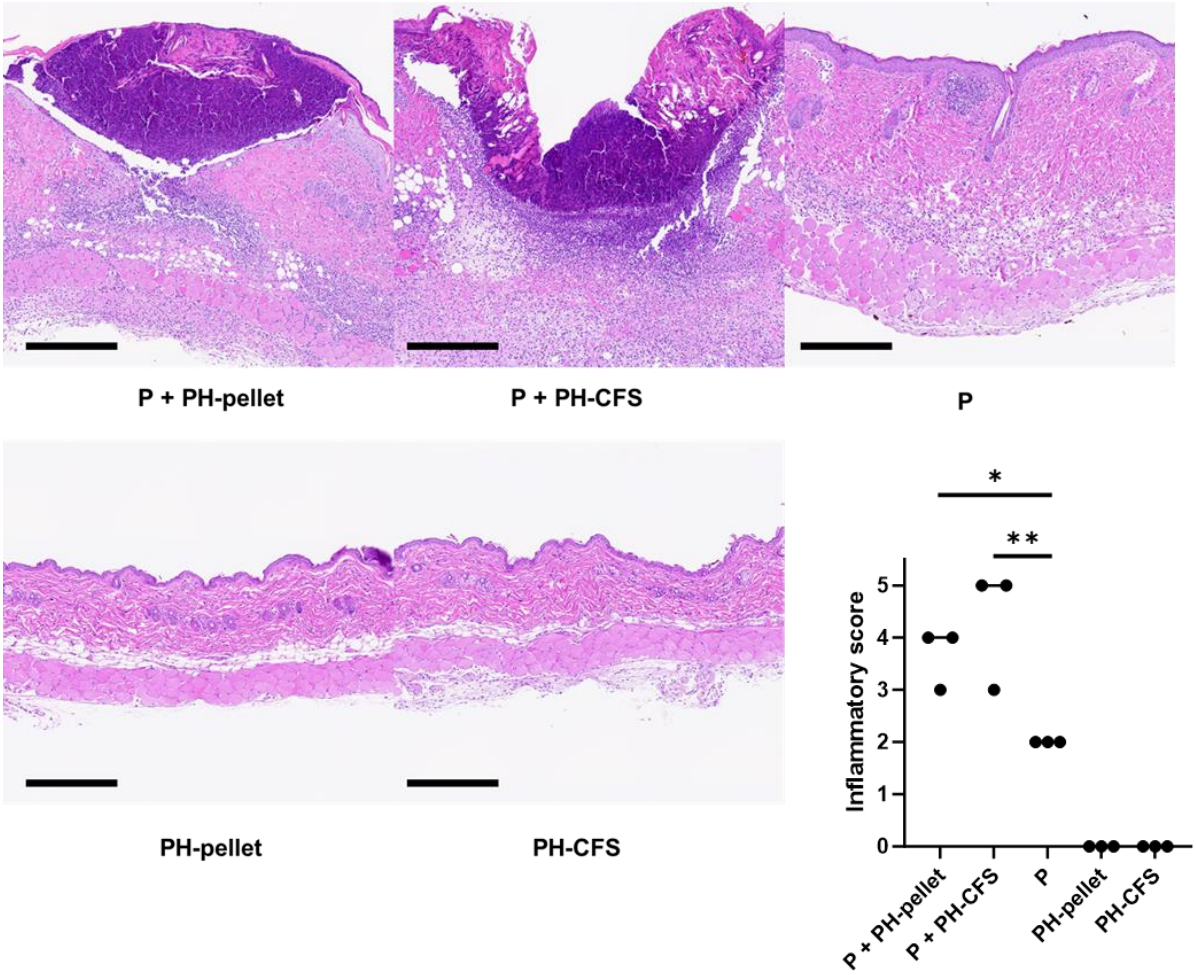

Supplementary Figure 3: Evaluation of histopathological lesions following pathogen and pathogen helper strain co-infection

Skin samples (n = 3) were evaluated 3 days after intradermal injection of the pathogen (P) or the pathogen helper strain (PH). The H&E stained images are representative of the histopathological characteristics of each group. Scale: 200 µm. The inflammatory score, measured based on the distribution and severity of infiltrated immune cells, is shown in the chart. Student's t-test: *$p < 0.05$, **$p < 0.01$ compared with the pathogen alone infected group.

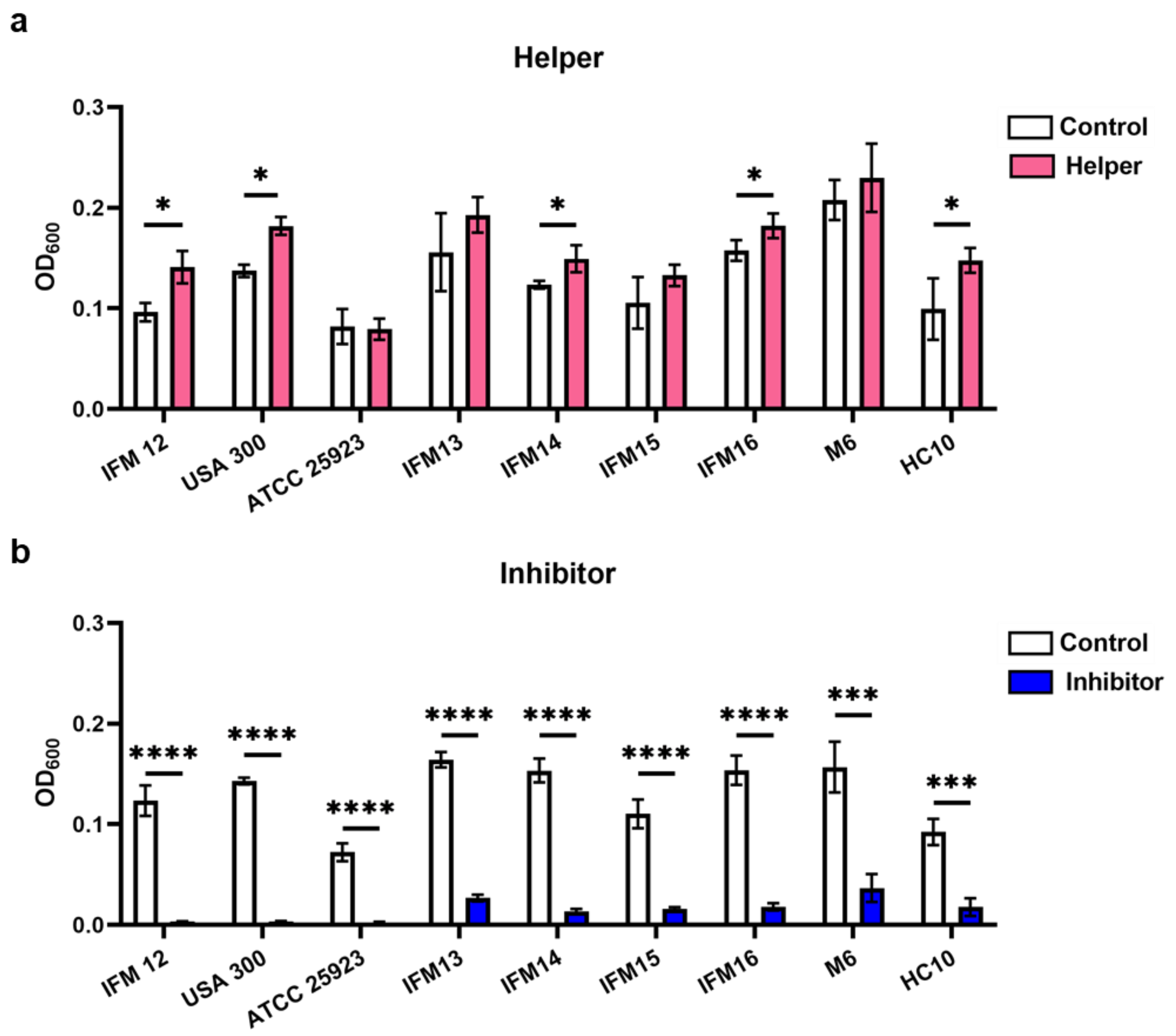

Supplementary Figure 4: Activities of pathogen helper and inhibitor bacteria on diverse *S. aureus* strains

Growth of nine *S. aureus* strains were measured after treatment with cell-free supernatant from pathogen helper (a) and inhibitor (b) bacteria. n = 5. Student's t-test: *, $p < 0.05$; ***, $p < 0.001$; ****, $p < 0.0001$ compared with the non-treated control.

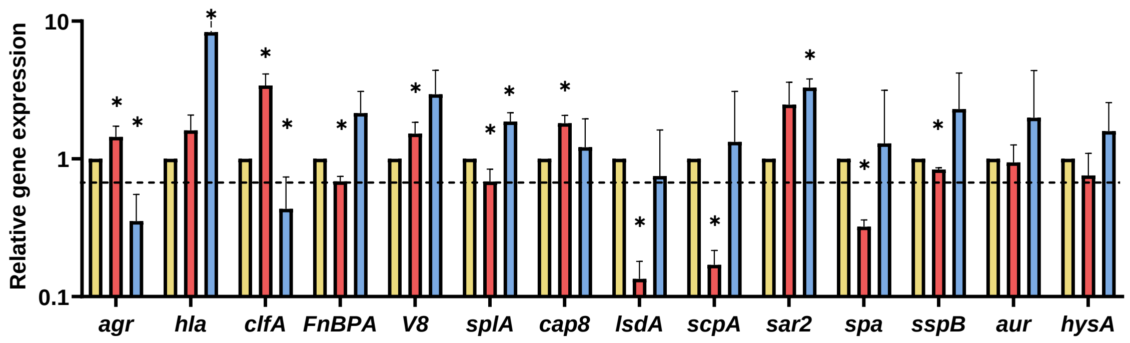

Supplementary Figure 5: Regulation of pathogen virulence factor expression by CFS treatment

Transcriptional changes induced by CFS in IFM12. qRT-PCR analysis of IFM12 gene expression following treatment with pathogen helper CFS and pathogen inhibitor CFS for 6 h. The data are expressed as the fold change in expression in treated versus nontreated IFM12. Student's t-test: *$p < 0.05$.

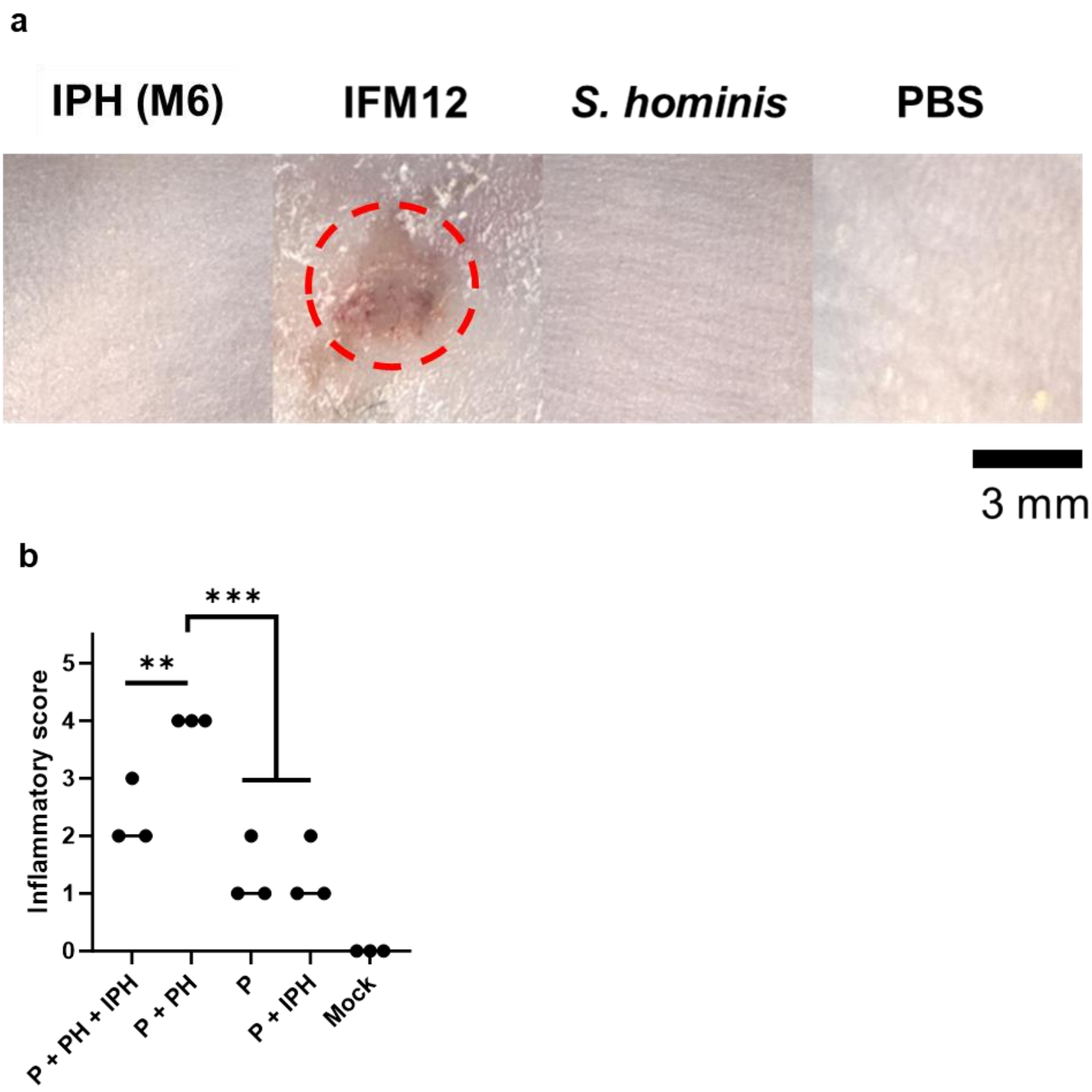

Supplementary Figure 6: Pathological evaluation for the IPH *S. aureus* M6-induced skin lesion.

a Representative images of skin lesions following intradermal infection with the IPH *S. aureus* M6, pathogenic *S. aureus* IFM12, and *S. hominis* negative control bacteria. The IFM12 infection site showed only a skin inflammatory lesion (red dotted circle). **b** Inflammatory score in the skin tissues when treated with IPH, PH, and pathogen. Student's t-test: $^{**}p < 0.01$, $^{***}p < 0.001$.

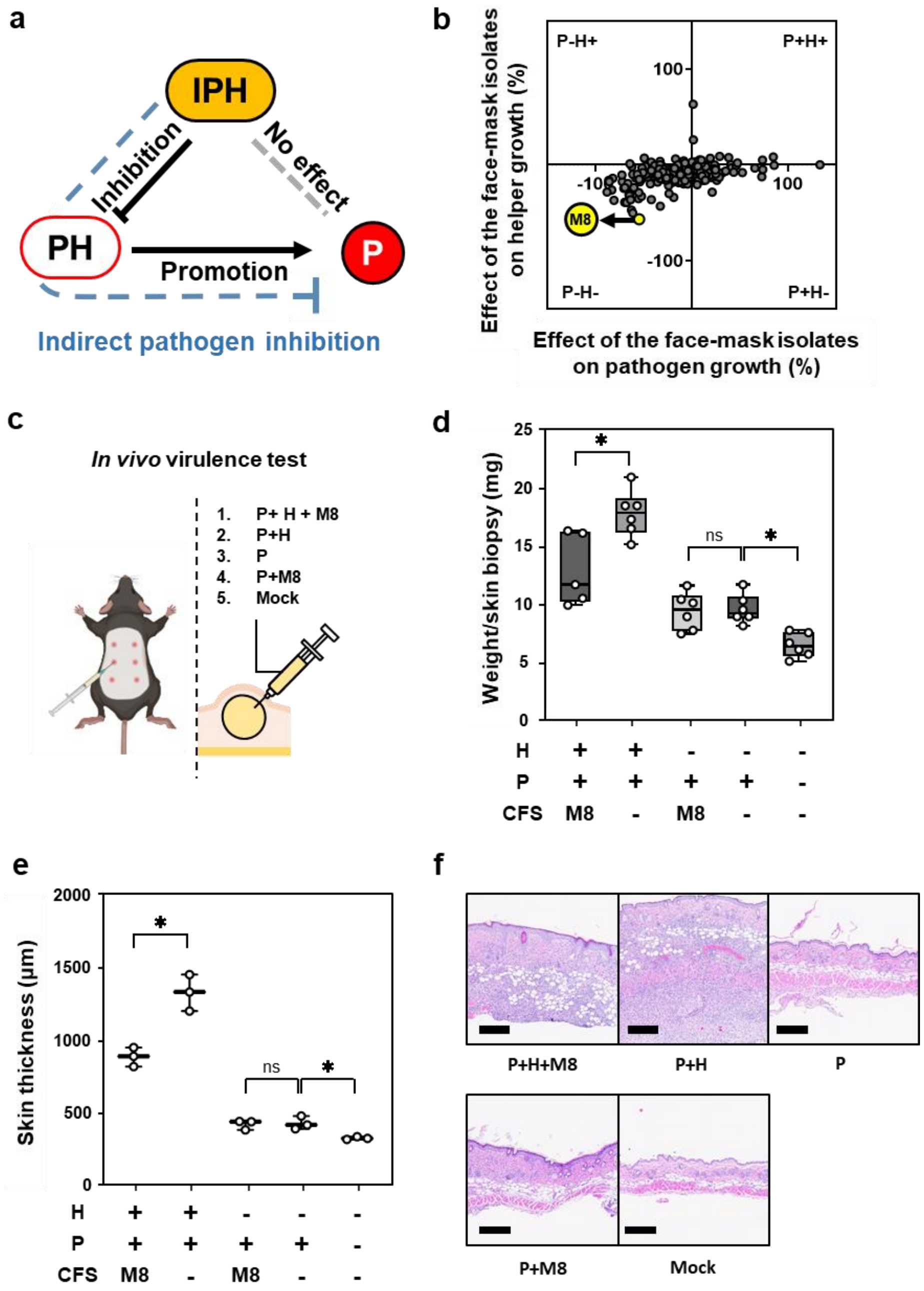

Supplementary Figure 7: Indirect interactions between skin virulent IFM and other IFM strains

a A diagram depicting direct and indirect effects of pathogen helper strains on pathogen growth. Inhibitor of pathogen helper strains (IPH) refer to any IFM isolate that indirectly inhibits pathogen growth by modulating pathogen helper strains. P, pathogen; PH, pathogen helper strain; IPH, inhibitor of pathogen helper strain. **b** Effects of conditioned supernatants from 200 IFM isolates on the growth of IFM12 and the pathogen helper strain *in vitro.* The x-axis shows the direct effect of each IFM isolate on IFM12. The y-axis shows the effect of each isolate on the pathogen helper strain. Of the 200 IFM culture supernatants

tested, M8 significantly inhibited growth of the pathogen and pathogen helper strain compared with media-treated controls. **c** Experimental schematic for intradermal infection. **d** Box plots depicting skin biopsy weight following pathogen infection in the presence or absence of the indirect inhibitor strain M8. **e** Box plots depicting skin thickness following pathogen infection in the presence or absence of the indirect inhibitor strain M8. Skin biopsies (n = 5) with a diameter of 5 mm were taken at 6 days after intradermal injection. The median, interquartile range, and all individual data points are shown. **f** Representative skin histopathological images with H&E stains. Scale bar = 500 µm.

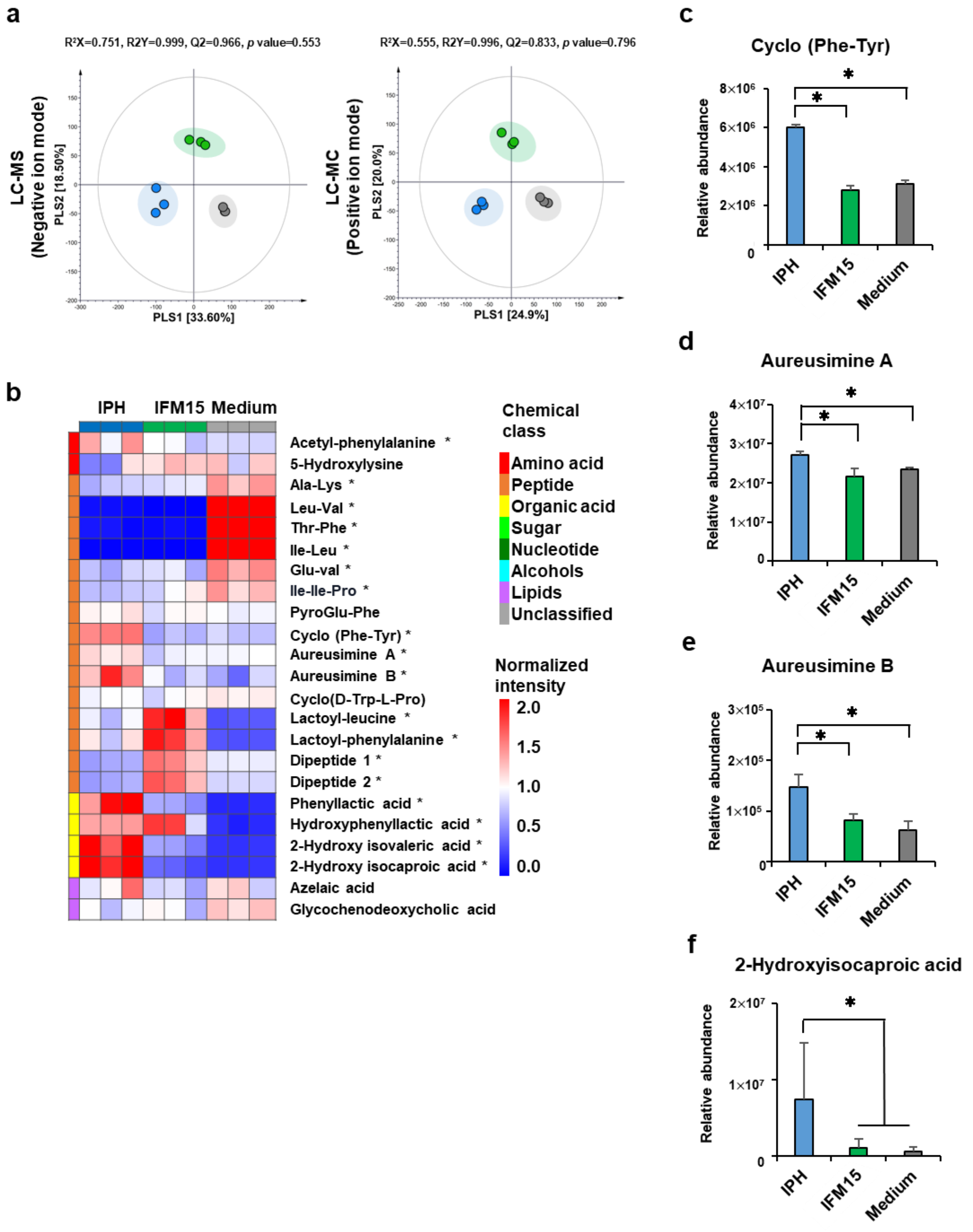

Supplementary Figure 8: Metabolic profiles analyzed by UHPLC-Orbitrap-MS to characterize CFS of IPH strain and IFM15 strain by fermentation

a Partial least squared discriminant analysis derived from UHPLC-Orbitrap-MS datasets. **b** Heatmap showing the average fold-change relative abundance of the discriminant metabolites between CFS and media control for IPH and IFM 15 strains in UHPLC-Orbitrap-MS datasets. **c-f** Bar charts shows the relative abundance of IPH-specific cyclic dipeptides derived from UHPLC-Orbitrap-MS. The discriminant metabolites in heatmaps were analyzed using a one-way ANOVA comparing 3: *$p < 0.05$. The IPH-specific

compounds showed significantly higher concentrations in the CFS of IPH strains than in the CFS of IFM 15 strain by fermentation. The asterisks in box charts denote significant dissimilarity between the metabolite of IPH strain and them of IFM15 strains or media control. Student's t-test: *$p < 0.05$ compared to CFS for IPH strain

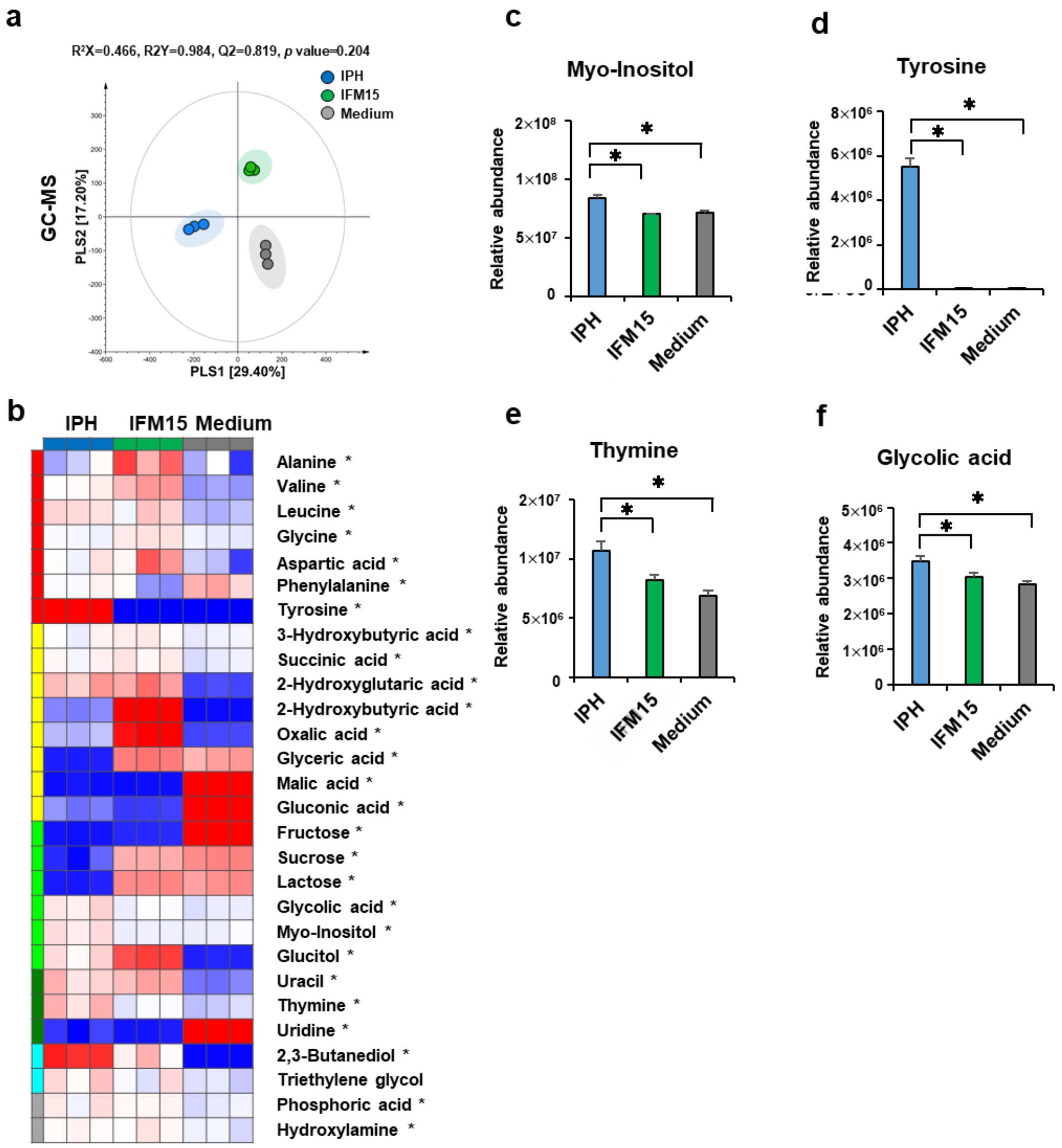

Supplementary Figure 9: Metabolic profiles analyzed by GC-TOF-MS to characterize CFS of IPH strain and IFM15 strain by fermentation

a Partial least squared discriminant analysis derived from GC-TOF-MS datasets and **b** Heatmap showing the average fold-change relative abundance of the discriminant metabolites between CFS and media control for IPH strain and IFM 15 strain in GC-TOF-MS dataset. **c-f** Box charts shows the relative abundance of IPH-specific compound derived from GC-TOF-MS. The discriminant metabolites in heatmaps were analyzed using a one-way ANOVA comparing 3: *$p < 0.05$. The IPH-specific compounds showed significantly higher concentrations in the CFS of IPH strains than in the CFS of IFM 15 strain by fermentation. The asterisks in box charts denote significant dissimilarity between the metabolite of IPH

strain and them of IFM15 strains or media control. Student's t-test: *$p < 0.05$ compared to CFS for IPH strains

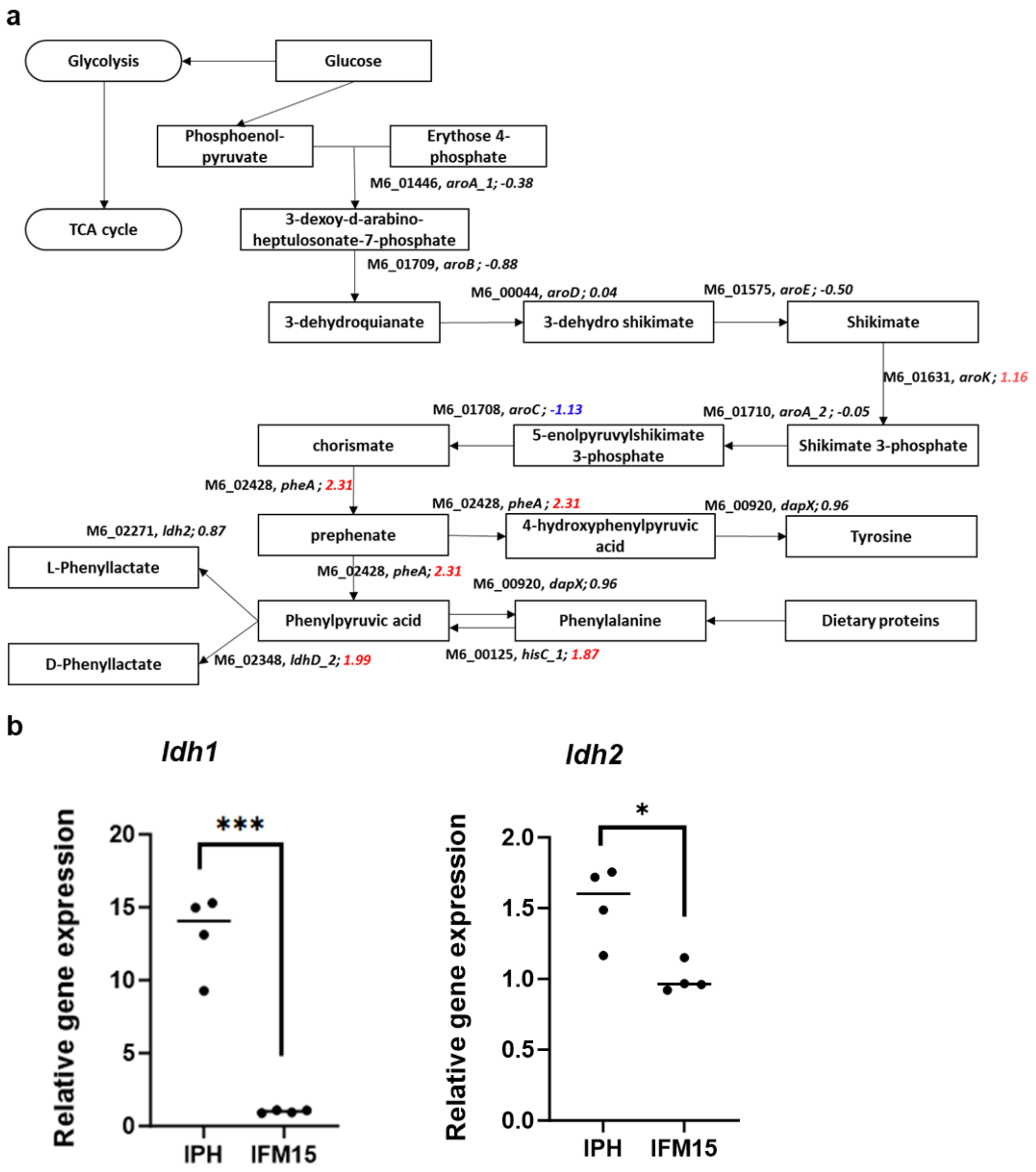

Supplementary Figure 10: Prediction of possible metabolic pathway associated with phenyllactic acid biosynthesis in IPH strain M6

a A diagram depicting the possible phenyllactic acid biosynthesis pathway of IPH strain M6. Red and blue number indicate upregulation and downregulation of genes with log2 fold change, respectively. **b** Validation for expression levels of genes associated with phenyllactic acid biosynthesis using quantitative PCR. Student's t-test: *$p < 0.05$, ***$p < 0.001$.
