# Supplementary material for: Facemask acne attenuation through modulation of indirect microbiome interactions
Source: NPJ Biofilms Microbiomes. 2024 Jun 20;10:50. doi: 10.1038/s41522-024-00512-w (PMC11190265; doi:10.1038/s41522-024-00512-w)
Supplement: Supplementary file 2 — Reporting summanry [file 41522_2024_512_MOESM2_ESM.pdf]

Corresponding author(s): Choong Min Ryu
Last updated by author(s): Mar 11, 2024

Reporting Summary

Nature Portfolio wishes to improve the reproducibility of the work that we publish. This form provides structure for consistency and transparency in reporting. For further information on Nature Portfolio policies, see our Editorial Policies and the Editorial Policy Checklist.

Statistics

For all statistical analyses, confirm that the following items are present in the figure legend, table legend, main text, or Methods section.

n/a	Confirmed	
☐	☒	The exact sample size (*n*) for each experimental group/condition, given as a discrete number and unit of measurement
☐	☒	A statement on whether measurements were taken from distinct samples or whether the same sample was measured repeatedly
☐	☒	The statistical test(s) used AND whether they are one- or two-sided<br>*Only common tests should be described solely by name; describe more complex techniques in the Methods section.*
☐	☒	A description of all covariates tested
☐	☒	A description of any assumptions or corrections, such as tests of normality and adjustment for multiple comparisons
☐	☒	A full description of the statistical parameters including central tendency (e.g. means) or other basic estimates (e.g. regression coefficient) AND variation (e.g. standard deviation) or associated estimates of uncertainty (e.g. confidence intervals)
☐	☒	For null hypothesis testing, the test statistic (e.g. *F*, *t*, *r*) with confidence intervals, effect sizes, degrees of freedom and *P* value noted<br>*Give P values as exact values whenever suitable.*
☒	☐	For Bayesian analysis, information on the choice of priors and Markov chain Monte Carlo settings
☒	☐	For hierarchical and complex designs, identification of the appropriate level for tests and full reporting of outcomes
☒	☐	Estimates of effect sizes (e.g. Cohen's *d*, Pearson's *r*), indicating how they were calculated

Our web collection on statistics for biologists contains articles on many of the points above.

Software and code

Policy information about availability of computer code

Data collection	No software was used to collect the data reported in this manuscript.
Data analysis	Statistical analyses and graphs were performed using GraphPad Prism version 10.

For manuscripts utilizing custom algorithms or software that are central to the research but not yet described in published literature, software must be made available to editors and reviewers. We strongly encourage code deposition in a community repository (e.g. GitHub). See the Nature Portfolio guidelines for submitting code & software for further information.

Data

Policy information about availability of data

All manuscripts must include a data availability statement. This statement should provide the following information, where applicable:

- Accession codes, unique identifiers, or web links for publicly available datasets
- A description of any restrictions on data availability
- For clinical datasets or third party data, please ensure that the statement adheres to our policy

The raw 16S rRNA gene sequence data produced as part of the present work were deposited at the NCBI SRA database (Accession number: PRJNA1058139). Additionally, RNA-seq data were deposited at the NCBI GEO database (Accession number: GSE248604)

Research involving human participants, their data, or biological material

Policy information about studies with human participants or human data. See also policy information about sex, gender (identity/presentation), and sexual orientation and race, ethnicity and racism.

Reporting on sex and gender	We used the term 'sex' throughout the manuscript to refer to the biological identity of the human participants.
Reporting on race, ethnicity, or other socially relevant groupings	N/A
Population characteristics	40 healthy volunteers of ages 20–59 years with comparable numbers of males and females were recruited. All volunteers self-reported to be in good general health with no underlying diseases
Recruitment	Participants were recruited following public IRB guidance, and all participants provided written informed consent.
Ethics oversight	This study was approved by the Public Institutional Review Board Designated by Ministry of Health and Welfare IRB (IRB no.: P01-202112-11-001)

Note that full information on the approval of the study protocol must also be provided in the manuscript.

Field-specific reporting

Please select the one below that is the best fit for your research. If you are not sure, read the appropriate sections before making your selection.

☒ Life sciences ☐ Behavioural & social sciences ☐ Ecological, evolutionary & environmental sciences

For a reference copy of the document with all sections, see nature.com/documents/nr-reporting-summary-flat.pdf

Life sciences study design

All studies must disclose on these points even when the disclosure is negative.

Sample size	sample size was determined by in basis on previously data with mean, standard deviation and effect size.
Data exclusions	No data exclusion except for sample loss.
Replication	in vitro data was validated through 2-3 replicates
Randomization	Animals used in this study were randomly distributed.
Blinding	This study is an observational study and no pre-determined groups were needed

Reporting for specific materials, systems and methods

We require information from authors about some types of materials, experimental systems and methods used in many studies. Here, indicate whether each material, system or method listed is relevant to your study. If you are not sure if a list item applies to your research, read the appropriate section before selecting a response.

Materials & experimental systems

n/a	Involved in the study	
☒	☐	Antibodies
☒	☐	Eukaryotic cell lines
☒	☐	Palaeontology and archaeology
☐	☒	Animals and other organisms
☐	☒	Clinical data
☒	☐	Dual use research of concern
☒	☐	Plants

Methods

n/a	Involved in the study	
☒	☐	ChIP-seq
☒	☐	Flow cytometry
☒	☐	MRI-based neuroimaging

Animals and other research organisms

Policy information about studies involving animals; ARRIVE guidelines recommended for reporting animal research, and Sex and Gender in Research

Laboratory animals	We used 7-10 female C57BL/6 mice
Wild animals	N/A
Reporting on sex	We used female animal because no different results between male and female was observed in previous experiment.
Field-collected samples	N/A
Ethics oversight	The experiments were conducted following the approved protocols by the Institute Animal Care and Use Committee of Korea Research Institute of Bioscience and Biotechnology (AEC-22118)

Note that full information on the approval of the study protocol must also be provided in the manuscript.

Clinical data

Policy information about clinical studies

All manuscripts should comply with the ICMJE guidelines for publication of clinical research and a completed CONSORT checklist must be included with all submissions.

Clinical trial registration	We are registering the study in Clinical Research Information Service (CRIS) with code P01-202112-11-001
Study protocol	Study protocol will be found in CRIS service with code P01-202112-11-001
Data collection	The data collection was performed from February 14, 2022, for 6 months
Outcomes	This study is an observational study. There are no evaluation end points.

Plants

Seed stocks	N/A
Novel plant genotypes	N/A
Authentication	N/A
